# Mitochondrial TSPO Deficiency Triggers Retrograde Signaling in MA-10 Mouse Tumor Leydig Cells

**DOI:** 10.3390/ijms22010252

**Published:** 2020-12-29

**Authors:** Jinjiang Fan, Vassilios Papadopoulos

**Affiliations:** 1 The Research Institute of the McGill University Health Centre, Montreal, QC H4A 3J1, Canada; Jinjiang.fan@mcgill.ca; 2 Department of Medicine, McGill University, Montreal, QC H4A 3J1, Canada; 3 Department of Pharmacology and Pharmaceutical Sciences, School of Pharmacy, University of Southern California, Los Angeles, CA 90089, USA

**Keywords:** translocator protein, genomic edition, mitochondria, retrograde signaling, calcium homeostasis, RNA-seq

## Abstract

The mitochondrial translocator protein (TSPO) has been shown to bind cholesterol with high affinity and is involved in mediating its availability for steroidogenesis. We recently reported that targeted *Tspo* gene deletion in MA-10 mouse tumor Leydig cells resulted in reduced cAMP-stimulated steroid formation and significant reduction in the mitochondrial membrane potential (ΔΨ_m_) compared to control cells. We hypothesized that ΔΨ_m_ reduction in the absence of TSPO probably reflects the dysregulation and/or maintenance failure of some basic mitochondrial function(s). To explore the consequences of TSPO depletion via CRISPR-Cas9-mediated deletion (indel) mutation in MA-10 cells, we assessed the transcriptome changes in TSPO-mutant versus wild-type (Wt) cells using RNA-seq. Gene expression profiles were validated using real-time PCR. We report herein that there are significant changes in nuclear gene expression in *Tspo* mutant versus Wt cells. The identified transcriptome changes were mapped to several signaling pathways including the regulation of membrane potential, calcium signaling, extracellular matrix, and phagocytosis. This is a retrograde signaling pathway from the mitochondria to the nucleus and is probably the result of changes in expression of several transcription factors, including key members of the NF-κB pathway. In conclusion, TSPO regulates nuclear gene expression through intracellular signaling. This is the first evidence of a compensatory response to the loss of TSPO with transcriptome changes at the cellular level.

## 1. Introduction

Translocator protein (TSPO) is an outer mitochondrial membrane (OMM) protein involved in multiple biological functions, including direct or indirect roles in mitochondrial cholesterol transport and steroid hormone biosynthesis, porphyrin transport and heme synthesis, apoptosis, cell proliferation, and anion transport [1]. TSPO is highly expressed in steroid-synthesizing tissues and binds with high affinity to cholesterol and other numerous compounds [2]. The role of TSPO in steroid biosynthesis has been extensively investigated and was found in multiple protein complexes involved in mitochondrial cholesterol transport, including in several animal models of disease [3,4,5,6]. Previously, we showed that TSPO deletion in mouse tumor MA-10 Leydig cells led to reduced mitochondrial membrane potential (ΔΨ_m_) and steroid biosynthesis under cAMP stimulation [7].

The effect of TSPO depletion on ΔΨ_m_ has been reported in mouse MA-10 cells [7], fibroblasts [8], human neuroblastoma cells [9] and mouse and rat cardiomyocytes [10,11]. Moreover, the dysregulation of the mitochondrial electron transport chain, such as up-regulated *Ucp2* or genes with similar function(s), seems to be a hallmark of TSPO deficiency in agreement with studies performed with various cell lines, including MA-10 cells, as well as in rodents [7,12]. This dysregulation has strong association with reduced proton leak [13], decreased ΔΨ_m_ [8], calcium homeostasis [9,14], and probably many other cellular events and responses [15]. There is only one report that failed to show any effect of TSPO on ΔΨ_m_ [12], and this could be attributed to the techniques used to measure ΔΨ_m_, since the report used an indirect measurement of ΔΨ_m_ using Förster resonance energy transfer between Mitotracker Green FM (MTG) and tetramethylrhodamine methyl ester (TMRM) [16]. In fact, the role of TSPO in ΔΨ_m_ was further confirmed by independent studies where TSPO depletion (knockout) in human primary microglia cells, as well as mouse BV2 cells and mouse primary microglia, led to decreased mitochondrial membrane potential, cytosolic Ca^2+^ homeostasis, and reduced respiratory function [17,18]. Moreover, TSPO was shown to be involved in the OMM-based pathway to control intracellular Ca^2+^ dynamics and redox transients in human SH-SY5Y neuroblastoma cells [9].

To study why the loss of TSPO has such as impact on basic mitochondrial function (ΔΨ_m_) and cell-specific roles (steroidogenesis) in more detail, we investigated gene expression profiles under TSPO deficiency via RNA-seq analysis. Interestingly, we report herein whole transcriptome changes in response to *Tspo* mutation, as well as showing that the top functional clusters of genes are related to extracellular matrix (ECM) proteins. We also found that the effected transcription factors, which may involve second messengers, such as cyclic nucleotides(e.g., cAMP), intracellular calcium ions (Ca^2+^) homeostasis, reactive oxygen species (ROS), might play an important role in retrograde signaling from the mitochondria to modulate the expression of nuclear genes.

## 2. Results

### 2.1. RNA-seq Data Production and the Verification of Tspo Gene Indel Deletion Mutation after CRISPR Gene Editing

**Figure 1:**
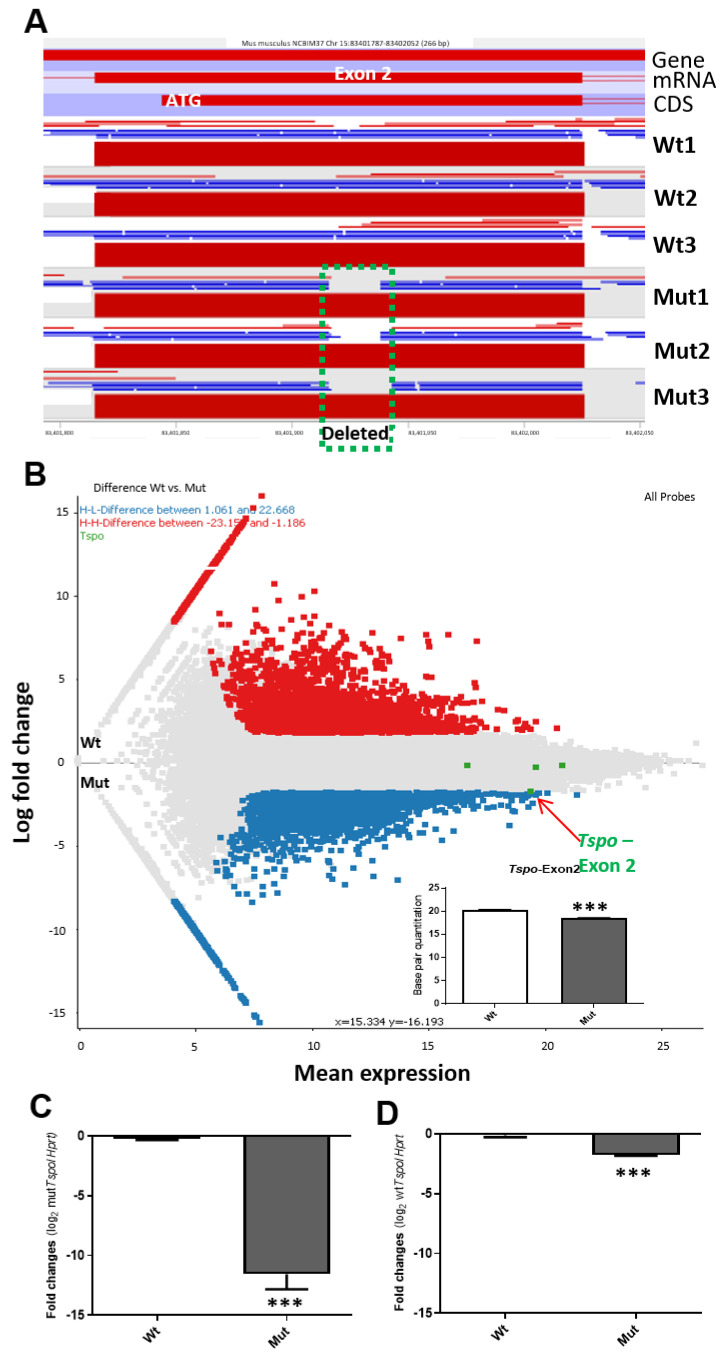
The genome mapping of *Tspo* exon2 after CRISPR/Cas9-mediated genome editing and the transcriptome changes under TSPO deficiency. (**A**). RNA-seq mapping of transcripts onto the *Tspo* exon 2. Gene, mRNA, CDS (coding sequence), and ATG are indicated. Deleted area is indicated as green square dotted line. Wt1, Wt2 and Wt3: three wild-type cell populations; Mut1, Mut2 and Mut3: three mutated cell line populations. (**B**). M-A plot of the comparison of RNA-seq data from Wt vs. Mut, by transforming the data differences onto M (log ratio, Y-axis) and A (mean average, x-axis) scales. Each exon of *Tspo* gene is indicated by green dots: exon2, where there is a small deletion, is indicated and shown to be significantly lower than that in Wt, using base pair quantitation as shown in bar graph. The rest of the exons show no significant differences. Each dot represents each exon; Red dots, highly expressed genes after TSPO depletion and blue dots, the less expressed genes after TSPO depletion; the grey dots are exons showing no dramatic changes between Mut and Wt cells. Inset, base pair quantitation of exon 2 of *Tspo* gene, which was partially deleted by the CRISPR/Cas9 genome editing. ***, *p* value < 0.001 (student *t*-test, *n* = 3). C–D. Real-time PCR of *Tspo* Mut transcripts using one of the primers located within the deleted area as shown in A (**C**) and the rest of the undeleted exon regions (**D**). ***, *p* value < 0.001 (student *t*-test, *n* = 3).

Total RNA samples from each *Tspo* mutant (Mut), as well as Wt cells, were sequenced using Illumina HiSeq4000 PE100 and then mapped to the mouse genome (NCBI Build 37). The *Tspo* locus was viewed in SeqMonk (http://www.bioinformatics.bbsrc.ac.uk/projects/seqmonk/), highlighting exon 2 where the deletions were made (Figure 1A). An MA plot was used to show the differential expression of whole transcripts, where each exon of *Tspo* mRNA was compared to show the lower expression of exon 2 with the deletions compared to the other three exons using base counting quantification (Figure 1B). Background expression of exon 2 was verified by real-time PCR using a targeted, deleted region-specific primer set (Figure 1C). The slightly reduced expression of the other three exons was also detected by real-time PCR using primer sets that do not target the deleted area (Figure 1D). Taken together, it is apparent that the indel mutation of the *Tspo* gene had been successfully introduced, but the expression levels of mutated *Tspo* RNA were about 11-fold lower, and the possible lower stability of full length, mutant mRNA(s) (about 1.6-fold difference).

**Figure 2:**
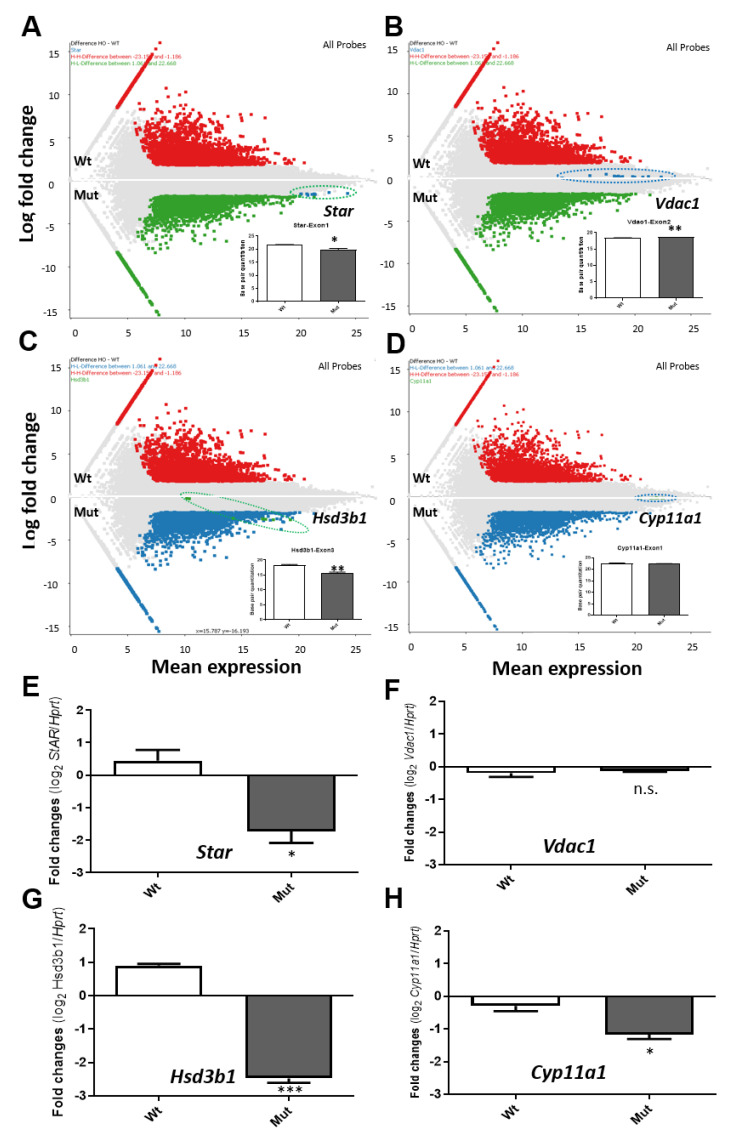
MA-plots of RNA-Seq data for selected important steroidogenic genes: *Star* (*Stard1*), *Vdac1*, *Hsd3b1*, and *Cyp11a1*. (**A**). Exons of *Star* are shown as blue dots, and the bar graph shows quantitation of the actual number of bases between Mut versus Wt. (**B**). Exons of *Vdac1* gene are shown as blue dots, and the bar graph shows the quantitation of the base difference between Mut versus Wt. (**C**). Exon of *Hsd3b1* gene are shown as green dots, and the bar graph shows the quantitation of the base difference between Mut versus Wt. (**D**). Exon of *Cyp11a1* gene are shown as green dots, and the bar graph shows the quantitation of the base difference between Mut versus Wt. (**E**,**H**). Real-time PCR of the four selected genes as shown in (**A**–**D**). Three steroidogenic genes *Star*, *Hsb3b1* and *Cyp11a1* are significantly down-regulated, whereas *Vdac1* shows no significant change. *, *p* value < 0.05 (student *t*-test, *n* = 3). **, *p* value < 0.01 (student *t*-test, *n* = 3). ***, *p* value < 0.001 (student *t*-test, *n* = 3).

The other genes important for steroidogenesis, *Star* (*Stard1*), *Vdac1*, *Hsd3b1*, and *Cyp11a1* were highlighted in whole transcripts in MA plots (Figure 2A–D), where the reduced levels of *Star* and *Hsd3b1* and the less or no change in transcription of *Vdac1* and *Cyp11a1*, respectively, were observed. It should also be noted that no significant changes were observed in *Vdac2* and *Vdac3* transcript levels in the absence of *Tspo* compared to control cells. The corresponding changes detected in RNA-seq data were all validated using real-time PCR (Figure 2E–H).

### 2.2. Transcriptome Changes after Tspo Mutation Affects the Extracellular Structure (ECS) and Extracellular Matrix (ECM)

Under TSPO deficiency, there is a transcriptome change, with a total of 8279 up-regulated and 6213 down-regulated genes (exons), yielding 463 genes up- and 778 genes down–regulated having values of base counts over 8 (Supplementary Materials Figure S1). To explore the interrogated functional associations among the up- or down-regulated genes expression, we used Metascape software for cross comparisons with numerous databases, such as GO Biological Processes, KEGG Pathways, and Reactome Gene Sets, to find enriched processes in the gene lists, as well as associations among enriched processes [19].

These Metascape results were dominated by functional categories related to control of the immune system, including humoral immune response, inflammatory response, complement activation, regulation of coagulation, regulation of proteolysis, and leukocyte mediated immunity, among others (Figures S2 and S3). A top functional annotation showed that up- and down-regulated genes were related to extracellular structure organization (ECS, Figure S2A, Table S1) and extracellular matrix organization (ECM, Figure S3A, Table S2), respectively. The corresponding gene networks were shown to indicate major biological functions (Figures S2B and S3B).

**Figure 3:**
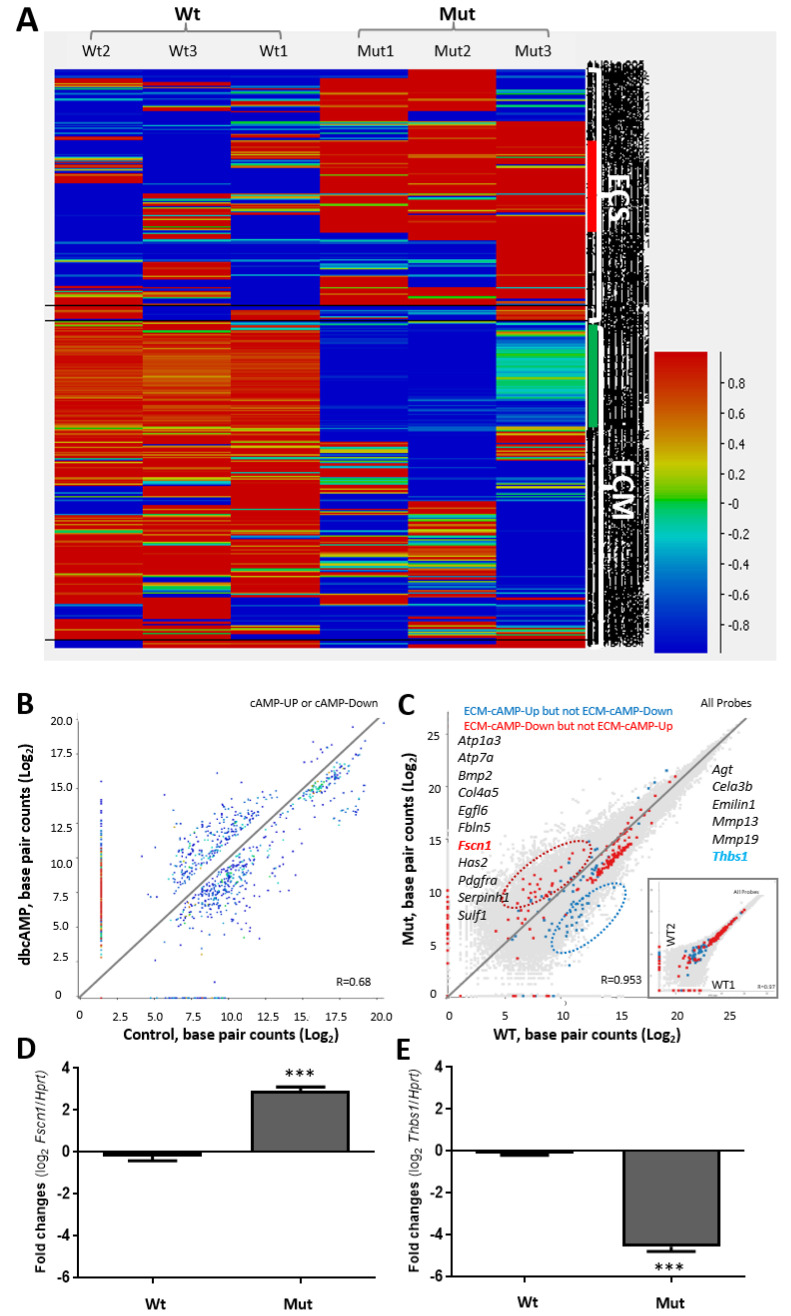
The disturbed expression of ECM and/or ECS genes after *Tspo* mutation in Mut cells. (**A**). Hierarchical cluster analysis of top functional groups of genes with either down- or up-regulation. The dramatic changes of the up-regulated extracellular structure organization (ECS) genes and the down-regulated extracellular matrix organization (ECM) genes are highlighted in hierarchical clustered heat maps from red (high) to blue (low), vertical bar. (**B**). Selected ECM and ECS genes in Wt cells from the hierarchical cluster analysis are distributed randomly in response to dbcAMP treatment. The diagonal line in the figure separates the gene expression further into high (up-left) or low (low-right) responses to dbcAMP. The colors of the dots correspond to the colors assigned to the genes from the hierarchical cluster analysis above. Color spots along either the x- or y- axis are unreadable exon(s). (**C**). Unique up- (in red) or low (in blue) expression of the ECM and ECS genes in response to TSPO depletion under dbcAMP treatment. Insert, the two Wt cell lines are compared. An example from either up- and down-regulated genes (Fscn1 vs. Thbs1) is highlighted. (**D**,**E**). Real-time PCR of the two genes: Fscn1 versus Thbs1. ***, *p* value < 0.001 (student *t*-test, *n* = 3).

A heat map shows that loss of TSPO altered genes related to either ECS or ECM, where ECS was up-regulated and ECM was down-regulated (Figure 3A). The expression of the genes from both groups were affected by treatment with the cell permeable cAMP analog dibutyryl-cAMP (dbcAMP) in Wt cells; either up-regulated (up-left of the diagonal line) or down-regulated (down-right of the diagonal line; Figure 3B). However, some of these genes, such as *Fscn1* and *Thbs1*, were actually disturbed by the *Tspo* mutation from comparison of Mut to Wt, where the red color represents the up-regulated and blue color represents the down-regulated genes from Figure 3B (Figure 3C). The changes were validated by one up-regulated gene (*Fscn1*) or one down-regulated gene (*Thbs1*) (Figure 3D,E). This suggests that ECS and/or ECM genes are related to steroidogenesis, which is consistence with previous reports [20,21].

### 2.3. Tspo Deletion Affects Transcriptome Changes, including Star

**Figure 4:**
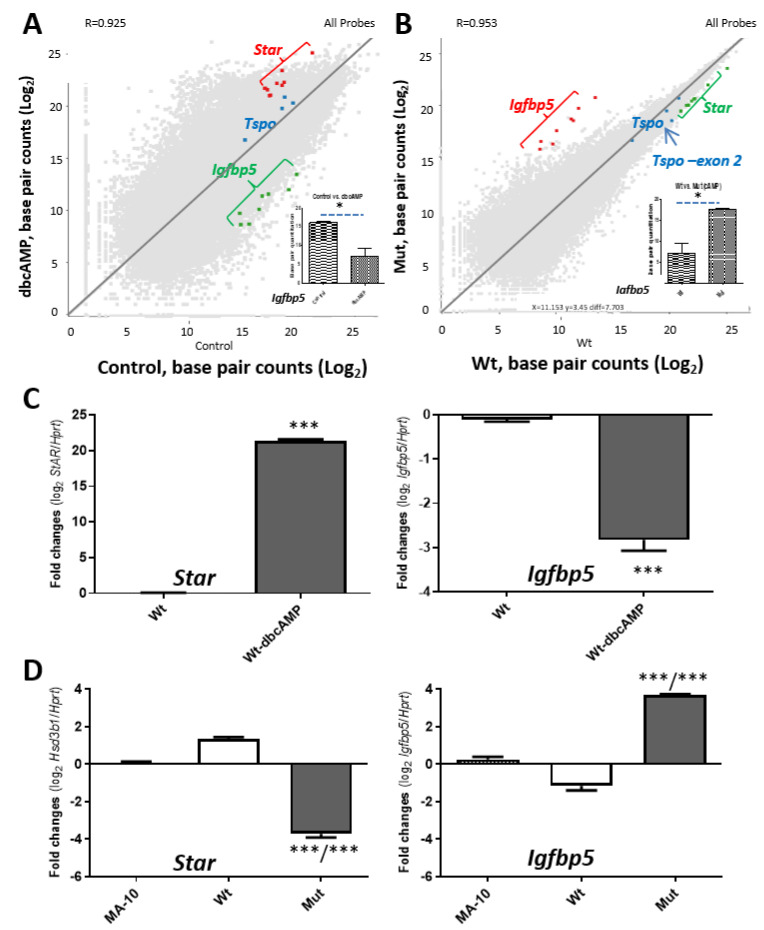
Possible compensatory mechanism to the loss of TSPO reflected in specific gene analysis. (**A**). A low expressed gene, insulin like growth factor binding protein 5 (*Igfbp5*), in comparisons with highly induced *Star* and slightly higher expressed *Tspo* in response to dbcAMP. (**B**). The opposite effect on three genes in response to *Tspo* mutation. The highly induced *Igfbp5* gene under TSPO depletion is highlighted, whereas *Star* and *Tspo* were used as gene expression control(s). Inset, the bar graph is the base pair quantitation of each exon of the *Igfbp5* gene. *, *p* value < 0.05 (Student *t*-test; *n* = 3). (**C**,**D**). Real-time PCR of *Star* and *Igfbp5* in response to dbcAMP stimulation in Wt cells (**C**) and to TSPO depletion in MA-10, Wt and Mut cells (**D**). ***, *p* value < 0.001 (student *t*-test, *n* = 3).

cAMP signal transduction pathway has been shown to be one of the major signaling cascades in steroidogenesis [22,23,24]. The dbcAMP-stimulated genes needed for steroidogenesis were disrupted under TSPO deficiency. In Wt MA-10 cells, *Star* levels were highly induced by cAMP treatment using dbcAMP, where insulin like growth factor binding protein 5 (*Igfbp5)* levels were reduced (Figure 4A). However, under the same stimulated condition, *Tspo* mutated cells contain high levels of *Igfbp5* and low levels of *Star*; the expression of *Tspo* is shown for reference (Figure 1B, Figure 2A and Figure 4B). The expression levels of both *Star* and *Igfbp5* under these conditions were verified using real-time PCR (Figure 4C,D).

### 2.4. Tspo Mutation Leads to Differential Expresion of Multiple Transcription Factors

**Figure 5:**
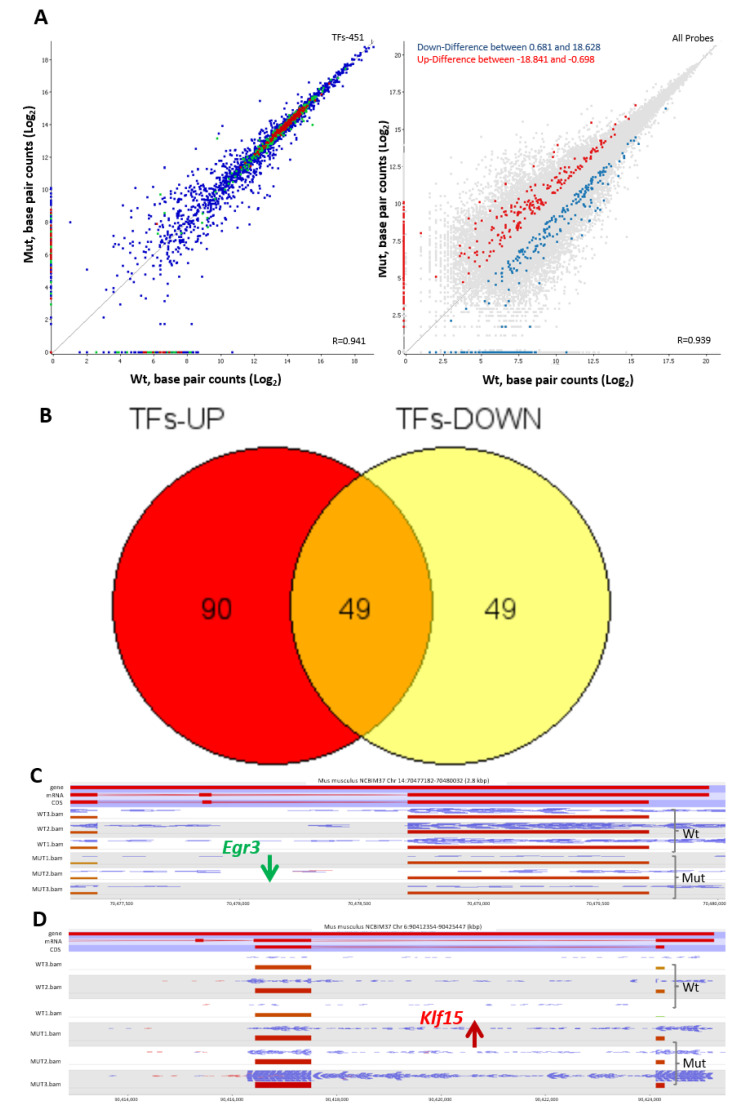
Transcription factors (TFs) affected by the loss of TSPO. (**A**). A scatter plot of a total of 451 mouse TFs from HOmo sapiens COmprehensive MOdel COllection (HOCOMOCO; http://hocomoco11.autosome.ru) between Wt vs. *Tspo* Mut (left) and their highlighted distribution in the total transcriptome changes (right). The color of the dots in the left panel represent TFs showing higher expression (red), reduced expression (green) and no change in expression levels (blue) between Wt and Mut cells. (**B**). 90 TFs are up-regulated and 49 TFs are down-regulated from a total of 188 TFs dramatically changed by the loss of TSPO. C-D. An example of down- (**C**) and up (**D**) regulated genes: *Egr3* (early growth response 3) and *Klf15* (Kruppel like factor 15), are presented. *Egr3* is an immediate-early growth response gene, which is induced by mitogenic stimulation, whereas *Klf15* is a negative regulator of TP53 acetylation that inhibits NF-κB activation through repression of EP300-dependent RELA acetylation.

The transcriptome changes due to the loss of TSPO were also reflected by changes in expression levels of many other transcription factors (TFs) [25]. A total of 451 TFs were investigated (Figure 5, left) and the up- and down-regulated TFs levels are shown in the whole RNA-seq data (Figure 5, right). There are 90 TFs up-regulated and 49 TFs down-regulated (Figure 5B; Table S3). The chromosomal view of two representative TFs: *Egr3* and *Klf15* are indicated in Figure 5C,D.

Affected TFs are involved in pattern specification and embryonic development, indicating the role of TSPO in cell fate decisions (Figure S4). Cell fate decision is under precise regulation by many factors, including the extracellular environment, cell-cell interactions, cell signaling, and cell metabolism, and all of these are related to mitochondrial energy metabolism, mitochondrial proteostasis, mitophagy, and key mitochondrial signaling events [26]. The loss of mitochondrial TSPO can reshape mitochondrial function, and the resulting imbalanced mitochondrial plasticity is reflected by changes in the nuclear transcriptional program.

### 2.5. Transcriptome Changes Due to the Loss of TSPO Are Related to the NF-κB Pathway

**Figure 6:**
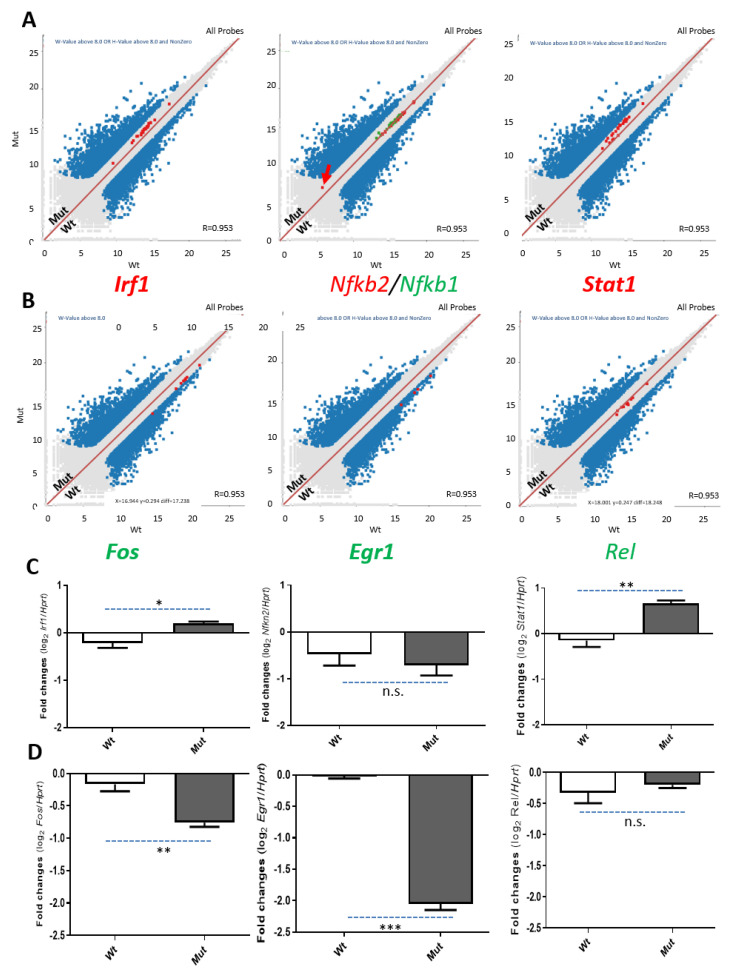
The NF-κB pathway is the link between the effects of the loss of TSPO on mitochondria and changes in nuclear gene expression. (**A**). Up-regulation of three genes involved in the NF-κB pathway: *Irf1*, interferon regulatory factor 1, and the whole set of *Irf1* exons are up-regulated as an early actin-rearrangement-inducing factor gene; Nfkb2/1, nuclear factor kappa B subunit 2/1, and one of the *Nfkb1* exons (exon 1, indicated in red arrow) are dramatically up-regulated, indicating there is an isoform-specific change; and *Stat1,* signal transducer and activator of transcription 1, and all of the *Stat1* exons are up-regulated as a whole gene. (**B**). Down-regulation of three genes involved in the NF-κB pathway: *Fos*, a proto-oncogene, AP-1 transcription factor subunit (with Jun); *Egr1*, early growth response protein 1; and *Rel*, REL proto-oncogene and NF-κB subunit. All of the genes from the NF-κB signaling pathways were selected from the NF-κB Signaling Pathway RT2 Profiler™ PCR Array PAMM-025Z (Qiagen, Germantown, MD, USA). (**C**,**D**). Real-time PCR of the six genes shown in A and B. Both *Irf1* and *Stat1* up-regulation and *Fos* and *Egr1* down-regulation in *Tspo* Mut cells were confirmed. n.s., non-significant; * *p* < 0.05, ** *p* < 0.01, and *** *p* < 0.001; Student’s *t*-tests (*n* = 3).

A set of genes that are responsible for the NF-κB pathway, selected from the enriched functional clusters of the impacted gene expression for verification, is involved in calcium signaling (Figure S2A) [27,28]. The levels of transcription factors, *Irf1*, *Nfkb1* and *Stat1* were found increased in RNA-seq data, whereas the levels of *Fos*, *Erg1* and *Rel* were reduced (Figure 6A,B). Expression levels of the transcripts were further confirmed by real-time PCR (Figure 6C,D).

## 3. Discussion

TSPO has been shown to affect ΔΨ_m_ [7], as well as steroid hormone biosynthesis [1,4,29]. In this report we have provided comprehensive evidence of transcriptome changes related to the loss of TSPO in MA-10 Leydig cells, probably due to reduced ΔΨ_m_ [7]. In fact, ΔΨ_m_ could affect a wide range of cellular functions, such as mitochondrial respiration, energy conversion, among others. CRISPR-Cas9-mediated *Tspo* gene knockout has been shown to alter respiration and cellular metabolism in human primary microglia cells [17]. These studies raise the question as to whether *Tspo* mutation can induce transcriptome changes that mediate effects on cell and tissue functions. In two independent studies differing results were reported for transcriptome changes with *Tspo* knockout/genetic deletion [30,31]. In the first study, *Tspo* global deletion was shown not to affect steroidogenic gene expression in adrenal glands, except for a short list of 18 genes, but the expression levels of the targeted gene *Tspo* were not presented [30]. Global deletion of *Tspo* in mice also showed no effect on lung gene expression profiles [31]. However, re-analysis of both the adrenal and lung datasets, using base-pair quantification of each exon as a basic analytic unit, clearly showed disturbed expression profiles of the target gene *Tspo*, as well as many other genes due to the loss of TSPO [32]. These findings raise the question as to why there are such discrepancies between reports. One reason is differences in the methodologies used to analyze the data [30,31,32]. However, there may be more than that. Once a gene is modified in the genome, the first reaction of cells or organisms is to activate DNA repair mechanisms, such as non-homologous end joining (NHEJ), and the corresponding down-stream responses then reflect the changes in target gene(s) [33,34]. Therefore, it has been proposed that it is better to use RNAi/knockdown, instead of genomic modification, to define a gene’s biological functions [35]. However, knockdown too can trigger a compensatory response, i.e., up-regulated expression of the compensating gene(s), leading to a mild or no phenotype, thus overshadowing the function derived from the absence of the target gene [35].

Nevertheless, genomic modification using CRISPR-Cas9 is a widely accepted approach to dissecting a gene’s role, which should be reflected in changes at the transcriptome level. Indeed, several genes related to fatty acid oxidation *Cd36*, *Cpt1a*, *Acadm*, *Acadl,* and *Hadha* were found to be affected by the loss of TSPO in MA-10 cells and verified by real-time PCR to be affected in mouse adrenal glands from *Tspo* KO mice as well [12]. However, none of the genes known to be related to defective lipid metabolism showed differential expression from an RNA-seq data analysis of mouse adrenal glands obtained from *Tspo* KO mice [12,30]. This discrepancy indicates that there are differences between cells and tissue responses, methodologies used for gene editing, and/or limitations at the level of RNA-seq data analysis. Interestingly, our RNA-seq data showed that, despite the fact that *Tspo* was interrupted, leading to the absence of TSPO protein in cells, aberrant *Tspo* transcripts were still present, although at reduced levels in the *Tspo* mutated cells. Indeed, the CRISPR-Cas9-based mutagenesis of a target gene could result in exon skipping, even with the presence of aberrant protein, and therefore, in addition to the target gene’s role, the impact on genome-editing outcomes also needs to be considered [36,37]. As to the *Tspo* gene deletion of a large genome DNA fragment, the new aberrant mRNA, resulting from the fusion of the rest of the exons, should be also detected by either RNA-seq or real-time PCR [30], even though there might be an important difference when using CRISP-Cas9 to introduce gene disruption via small insertions/deletions versus gene deletion of large DNA fragments.

Expression of some other genes involved in the regulation of steroidogenesis, such as *Star*, was also affected in *Tspo* mutated cells. This was also shown in adrenal glands from *Tspo* KO mice [32]. In the same fashion, many other genes behave the same as we showed herein at the transcriptome level. The top two identified functional clusters from the whole transcriptome are related to ECS/ECM. Both groups of genes are involved in intercellular or extracellular matrices, which may be related the morphology of the cells. It has been reported that ECM is important for steroidogenesis [20,38,39]. Leydig cells reside in the interstitium, where there is abundant ECM affecting their shape, association, proliferation, and gene expression [38,40]. Moreover, the effect of pituitary hormone LH on Leydig cells depends on the ECM environment and alters the steroidogenic ability of the cells [39]. Other transcriptome changes could contribute to the multiple outcomes resulting from TPSO deficiency.

A signal transduction pathway, coined as mitochondrial retrograde signaling, was shown to convey information on the developmental and physiological state of mitochondria to the nucleus, leading to changes in nuclear gene expression [41]. The communication between mitochondria and the nucleus is reflected not only through normal mitochondrial biogenesis and activity, but also from mitochondrial malfunctions that trigger compensatory responses in the nucleus via different effectors, molecules and outcomes [41]. In mammalian cells, calcium sensitive signaling pathways have been proposed to play such a role by activating the NF-κB pathway at both the nuclear translocation and transcription levels [27,42,43,44,45]. Several key transcription factors in the NF-κB pathway, such as Irf1, Stat1, Fos and Egr1, were shown to be affected by the loss of TSPO in MA-10 cells. Indeed, data from both RNA-seq and real-time PCR support this conclusion.

Mitochondria are the largest cellular organelles involved in the synthesis and folding of membrane/secretory proteins, lipid metabolism and calcium storage [46]. Mitochondrial retrograde signaling is triggered by a mitochondrial signal(s) and allows the organelle to control nuclear gene transcription [47,48]. TSPO, as an evolutionarily conserved protein, must possess some conserved functions, such as ΔΨ_m_, to trigger the retrograde signaling leading to variable outcomes, including steroidogenesis [49] and other functions [50,51]. In addition to a signaling mechanism, direct mitochondria-nucleus interactions may drive the mitochondrial retrograde response. In 1999, we reported that the high TSPO expression levels in aggressive breast cancer cells correlated with perinuclear/nuclear localization of TSPO, TSPO-mediated cholesterol transfer into nucleus and increased cell proliferation [52]. These findings were recently confirmed and extended to demonstrate that TSPO is required for the formation of mitochondria-nucleus contact sites during the mitochondrial retrograde response [53]. This response allows for redistribution of cholesterol that deacetylates NF-kB thus completing the prosurvival response in aggressive breast cancer cells [53].

The data presented here focus on the role of the mitochondrial TSPO protein in retrograde signaling. The role of TSPO in mitochondria has been extensively studied using drug ligands specific for TSPO and shown to be involved in the regulation of mitochondrial membrane potential, calcium release, ATP production, ROS generation and cellular oxygen sensing [1,2,49,54]. Elevated TSPO expression has been linked to neurodegenerative disorders, such as Parkinson’s disease (PD) [55], as well as heart failure [11]. The common feature of these links are related to mitochondrial calcium and the production of ROS, where coordinated regulation of the calcium and ROS signaling systems seems to have important implications for fine tuning cellular signaling networks [56]. Calcium release, ATP production and ROS are part of the retrograde mitochondria-nucleus signaling pathway. Interestingly, Gavish and colleagues recently showed that TSPO drug ligands modulate nuclear gene expression, in addition to directly increasing TSPO expression levels, thus further implicating TSPO in mitochondrial retrograde signaling [57,58]. Our work supports these findings and extends them by demonstrating that TSPO itself is part of the mitochondrial–nuclear signaling pathway regulating nuclear gene expression. These functional changes could be driven by changes in several transcription factors affected by *Tspo* deletion, some of which may lead to cell fate decisions throughout the stages of preimplantation development in mouse embryos [32]. Although our work is focused on steroidogenesis, the implication of our findings includes other functions where TSPO has been shown to play important roles, such as cell growth and differentiation, apoptosis, inflammation and immune response.

In conclusion, we have described mitochondrial TSPO-dependent transcriptome changes in MA-10 Leydig cells. This study provides evidence on how intracellular retrograde signals are transmitted from mitochondria to the nucleus. Moreover, this study raises the question on how transcriptome changes induced by TSPO mutations might affect mitochondrial function (anterograde signaling).

## 4. Materials and Methods

### 4.1. Cell Culture

MA-10 cells were maintained in Dulbecco’s modified Eagle medium/F-12 medium (DMEM/F12; Gibco^®^, Thermo Fisher Scientific, Burlington, ON, Canada), supplemented with 5% heat-inactivated fetal bovine serum and 2.5% horse serum [59]. The MA-10-derived wild-type subcell lines (Wt, previously G1) and the *Tspo* genome-edited subcell lines (Mut, previously nG1) were the same as described previously [7]. In brief, the *Tspo* mutant cells were created via CRISPR-Cas9 system, and all cells were grown in this medium supplemented with 400 μg/mL of G418 (Roche Diagnostics, Indianapolis, IN, USA), 100 U/mL of penicillin, and 100 μg/mL of streptomycin in 5% CO_2_/air at 37 °C.

### 4.2. RNA-seq Data Generation and Data Analysis

The cells used for total RNA extraction were cultured in 6-well plates to 80% confluence. Total RNA samples from Wt and *Tspo* mutant cells were extracted using TRIzol reagent (Invitrogen; Thermo Fisher Scientific, Waltham, MA, USA) and then quantified with an Agilent 2100 bioanalyzer. The quantified RNA samples were used for NEBNext^®^ (New England Biolabs, Ipswich, MA, USA) rRNA-depleted (Human/Mouse/Rat) stranded library preparation. Total transcripts of the cells were sequenced at McGill University and the Genome Quebec Innovation Center. The original data reported in this paper have been deposited in the NCBI Gene Expression Omnibus database (GEO): accession no.: #####. Data analysis was performed using the RNA-Seq analysis pipeline in the Genome Analysis ToolKit from the Center. The RNA-Seq data was mapped to mouse genome (NCBIM37), analyzed, presented using the SeqMonk software package (http://www.bioinformatics.bbsrc.ac.uk/projects/seqmonk/; Babraham Bioinformatics, Cambridge, UK) and visualized in the UCSC Genome Browser (http://genome.ucsc.edu) with a custom track. Functional gene clustering was analyzed using MetaScape software (http://metascape.org) [19].

### 4.3. Real-Time PCR

Total RNA samples from Wt and *Tspo* mutant cells were extracted using TRIzol reagent (Invitrogen, Carslbad, CA, USA), then subject to deoxyribonuclease treatment and a removal reagent (DNA free; Ambion, Austin, TX, USA) before cDNA synthesis. Sample RNAs were subsequently diluted to 100 ng/μL using deoxyribonuclease/ribonuclease-free water, and first-strand cDNA was synthesized using the Transcriptor First Strand cDNA Synthesis Kit using the manufacturer’s protocol (Roche Life Science, Laval, QC, Canada). The resulting cDNA samples were diluted with nuclease-free water and subjected to real-time PCR using gene–specific primers (Table 1) and SYBR green dye in a LightCycler 480 system (Roche Life Science) as previously described [60]. The results reported for each RNA product were normalized to hypoxanthine-guanine phosphoribosyltransferase (*Hprt*) to correct for differences in the amount of the template cDNA.

### 4.4. Statistical Analysis

Data were expressed as mean ± standard error of the mean, and graphic presentation was performed using GraphPad Prism (version 5.02; GraphPad Software) for Windows. Statistical analyses of data were performed by the unpaired Student t test. Mean differences were considered statistically different when *p* < 0.05.

## 5. Conclusions

In this report, we have shown that TSPO pays an important role in maintaining the integrity of mitochondrial functions. Loss of the mitochondrial TSPO could lead to transcriptome changes via unidentified second massager(s)-mediated retrograde signaling.

## Figures and Tables

**Table 1:** Oligonucleotides used in this study.

Primers	Sequence	Purpose
Cyp11a1-F	CGCCATCACCTCTTGGTTTA	Real-time PCR
Cyp11a1-R	GGTGGCCTATCACCAGTATTATC	Real-time PCR
Cyp11b1-F	CGGCAACATCACAGATACGA	Real-time PCR
Cyp11b1-R	CAGGAACAAGTGGCTGAAGA	Real-time PCR
Dnahc5-F	CTGAGAGCTGGGATGAAGATG	Real-time PCR
Dnahc5-R	AGCCATTACGCCTGAGAAC	Real-time PCR
Egr1-F	GAGTCGTTTGGCTGGGATAA	Real-time PCR
Egr1-R	AACAACCCTATGAGCACCTG	Real-time PCR
Fos-F	GCAACGCAGACTTCTCATCT	Real-time PCR
Fos-R	GAATCCGAAGGGAACGGAATAA	Real-time PCR
Fscn1-F	TCTCCTGATCGGTCTCTTCATC	Real-time PCR
Fscn1-R	TCTTCGCCCTGGAACAGA	Real-time PCR
Hprt-F	GAGCAAGTCTTTCAGTCCTGTCCA	Real-time PCR
Hprt-R	GCGTCGTGATTAGCGATGATGAAC	Real-time PCR
Hsd3b1-F	CTGTCACCTTGGTCTTTGTCT	Real-time PCR
Hsd3b1-R	TGGTGCAGGAGAAAGAACTG	Real-time PCR
Hspb8-F	CTTCTGCAGGGAGCTGTATTT	Real-time PCR
Hspb8-R	CCGGAAGAGCTGATGGTAAAG	Real-time PCR
Igfbp5-F	CAGCCTTCAGCTCGGAAAT	Real-time PCR
Igfbp5-R	GGCGAGCAAACCAAGATAGA	Real-time PCR
Irf1-F	TTAGTGTCTCGGCTGGACTT	Real-time PCR
Irf1-R	AGGATCAGAGTAGGAACAAGGG	Real-time PCR
Nfkb2-F	GATCCTTAGGCTCCACGATG	Real-time PCR
Nfkb2-R	GGGCCTAGCCCAGAGATA	Real-time PCR
Nrxn1-F	GTAGCCCGTTGTGGTAAGAA	Real-time PCR
Nrxn1-R	ATGTGAAAGTCACCAGGAATCT	Real-time PCR
Psd4-F	GGCAGTTAGGCTCCAGTAAA	Real-time PCR
Psd4-R	CAGGTCCCAGGAAACTCTTATC	Real-time PCR
Rel-F	AACACAGCCTCACCACATT	Real-time PCR
Rel-R	GATTAGTGCAGGAATCAATCCTTTC	Real-time PCR
Star-F	ACTCTATCTGGGTCTGCGATA	Real-time PCR
Star-R	GGAAGTCCCTCCAAGACTAAAC	Real-time PCR
Stat1-F	GGTCGCAAACGAGACATCATA	Real-time PCR
Stat1-R	CCCATGGAAATCAGACAGTACC	Real-time PCR
Thbs1-R	CTAGGTGTCCTGTTCCTGTTG	Real-time PCR
Thbs-F	CACCTCCAATGAGTTCAAAGATG	Real-time PCR
Tspo-wt-F	TGTGAAACCTCCCAGCTCTTTCCA	Real-time PCR-WT
Tspo-wt-R	TAGCTTGCAGAAACCCTCTTGGCA	Real-time PCR-WT
Tspo-mut-F	CCCATGGCTGAATACAGTGTG	Real-time PCR-Deletion
Tspo-mut-R	GGGAGCCTACTTTGTACGTG	Real-time PCR-Deletion
Vdac1-F	AGTCCATCTGTACTTGGTTTCC	Real-time PCR
Vdac1-R	ACGAAGTCAGAGAATGGATTGG	Real-time PCR

## Data Availability

The data presented in this study are available in the manuscript, the supplementary material, and the NCBI Gene Expression Omnibus database (GEO): accession no.: #####.

## Supplementary Materials

Supplementary materials can be found at https://www.mdpi.com/1422-0067/22/1/252/s1.

## Abbreviations

TSPO	translocator protein
CRISPR/Cas9	clustered regularly interspaced short palindromic repeats/ CRISPR-associated protein 9
dbcAMP	dibutyryl-cAMP
ΔΨ_m_	mitochondrial membrane potential
Wt	wild-type
PCR	polymerase chain reaction
RNA-Seq	RNA sequencing
ECM	extracellular matrix
ECS	extracellular structure organization
EGTA	ethylene glycol-bis(β-aminoethyl ether)-N,N,N′,N′-tetraacetic acid
Ca^2+^	Calcium ion
