# Supplementary material for: Mitochondrial TSPO Deficiency Triggers Retrograde Signaling in MA-10 Mouse Tumor Leydig Cells
Source: Int J Mol Sci. 2020 Dec 29;22(1):252. doi: 10.3390/ijms22010252 (PMC7795497; doi:10.3390/ijms22010252)
Supplement: Supplementary file 1 [file ijms-22-00252-s001.zip › ijms-991545-supplementary-Figure.pdf]

Supplementary Materials

Mitochondrial TSPO Deficiency Triggers Retrograde Signaling in MA-10 Mouse Tumor Leydig Cells

Jinjiang Fan [1,2] and Vassilios Papadopoulos [1,2,3,*]

Supplementary Figures

Figure. S1. A scatter plot of each exon from base pair counting quantification to show transcriptome changes revealed from RNA-seq data under TSPO deficiency.

Figure. S2. Overview of enriched clusters in GO terms obtained from RNA-seq analysis of up-regulated mRNA expression from *Tspo* mutated MA-10 cells

Figure. S3. Overview of enriched clusters in GO terms obtained from RNA-seq analysis of down-regulated mRNA expression in *Tspo* mutated MA-10 cells.

Figure S4. An UP (A) and DOWN (B) regulated gene network due to the loss of TSPO.

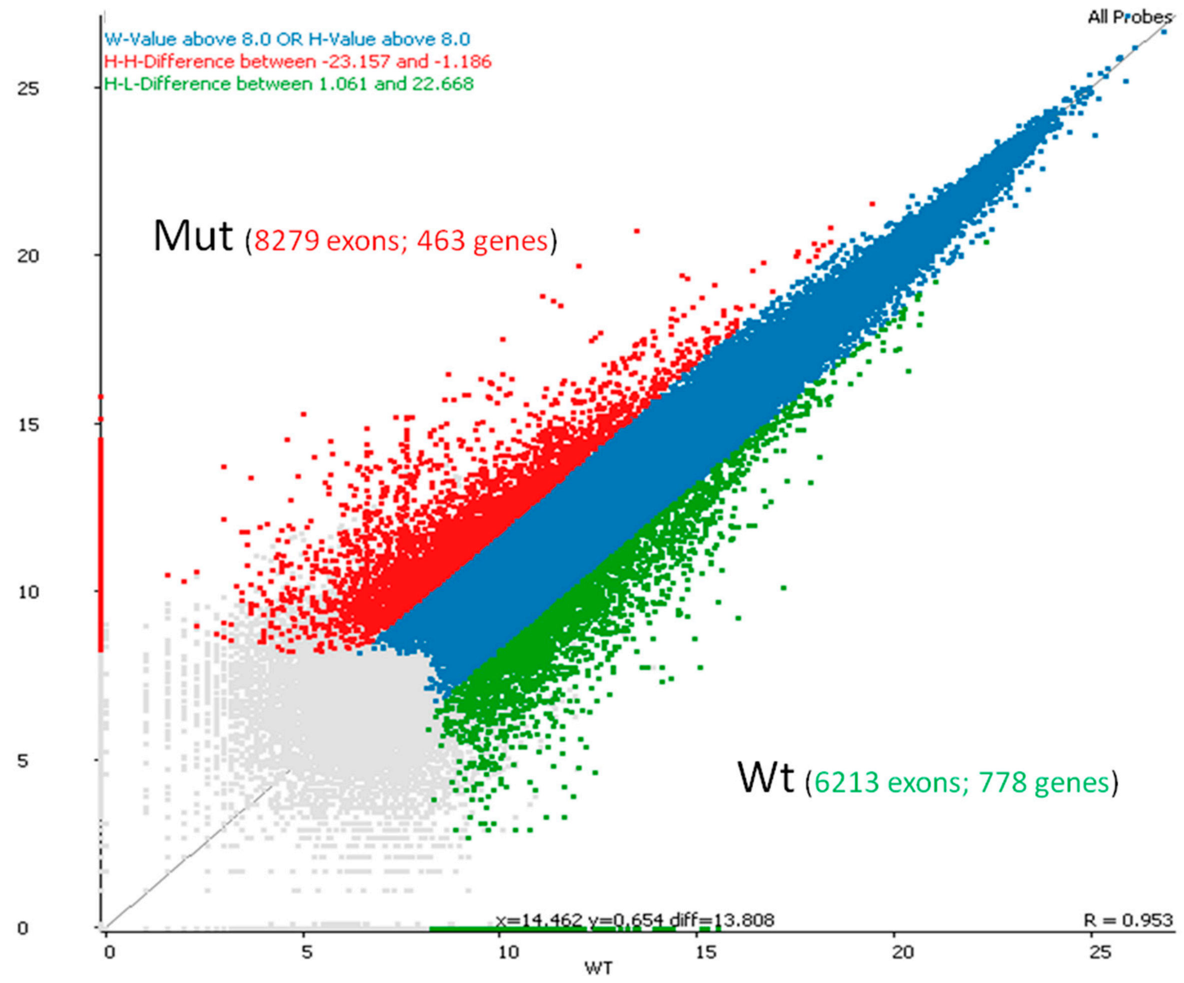

Figure S1. A scatter plot of each exon from base pair counting quantification to show the transcriptome changes under TSPO deletion mutation revealed from RNA-seq data. As previously reported on expression changes of a large number of genes from re-analysis of two data sets of RNA-seq from animal studies with *Tspo* global deletion or mutation, our data indicate transcriptome changes at the cellular level in response to the TSPO deficiency. Red spots, 463 up-regulated exons, which represent either single exon or a group of exons from a single gene; green, 778 down-regulated single exons or a group of exons from a single gene. The exons with less dramatic changes in expression are depicted as blue. Wt vs. Mut.

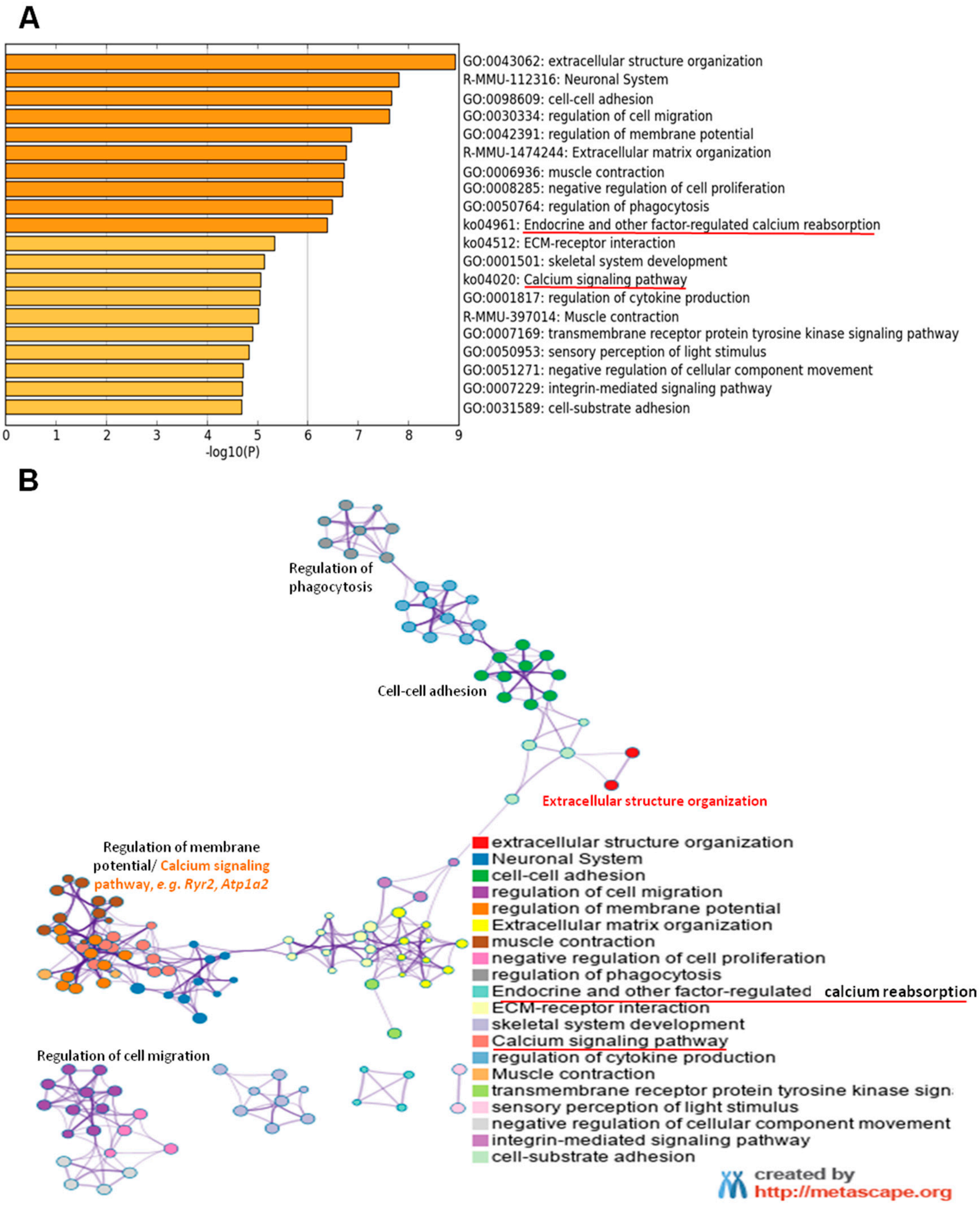

Figure S2. Overview of enriched clusters in GO terms obtained from RNA-seq analysis of up-regulated mRNA expression from *Tspo* mutated MA-10 cells. Statistically significant enriched terms (GO/KEGG terms and/or canonical pathways) were identified using Metascape (http://metascape.org). Accumulative hypergeometric p-values and enrichment factors were calculated and used for filtering. Remaining significant terms were hierarchically clustered into a tree based on Kappa statistical similarities among gene memberships. A Kappa score of 0.3 was applied as the threshold to cast the tree into term clusters. We selected a term with the lowest p-value as the representative term for each cluster and show the p-values of all clusters in the bar graphs. A. Cluster analysis for up-regulated genes under TSPO mutation. B. Enriched pathways from the up-regulated mRNA expression in *Tspo* muted MA-10 cells. Gene-set clustering maps were generated using MetScape 3 for Cytoscape. The most significant shifting was the up-regulation of extracellular structure organization (ECS), cell-cell adhesion, regulation of phagocytosis and calcium signaling pathway.

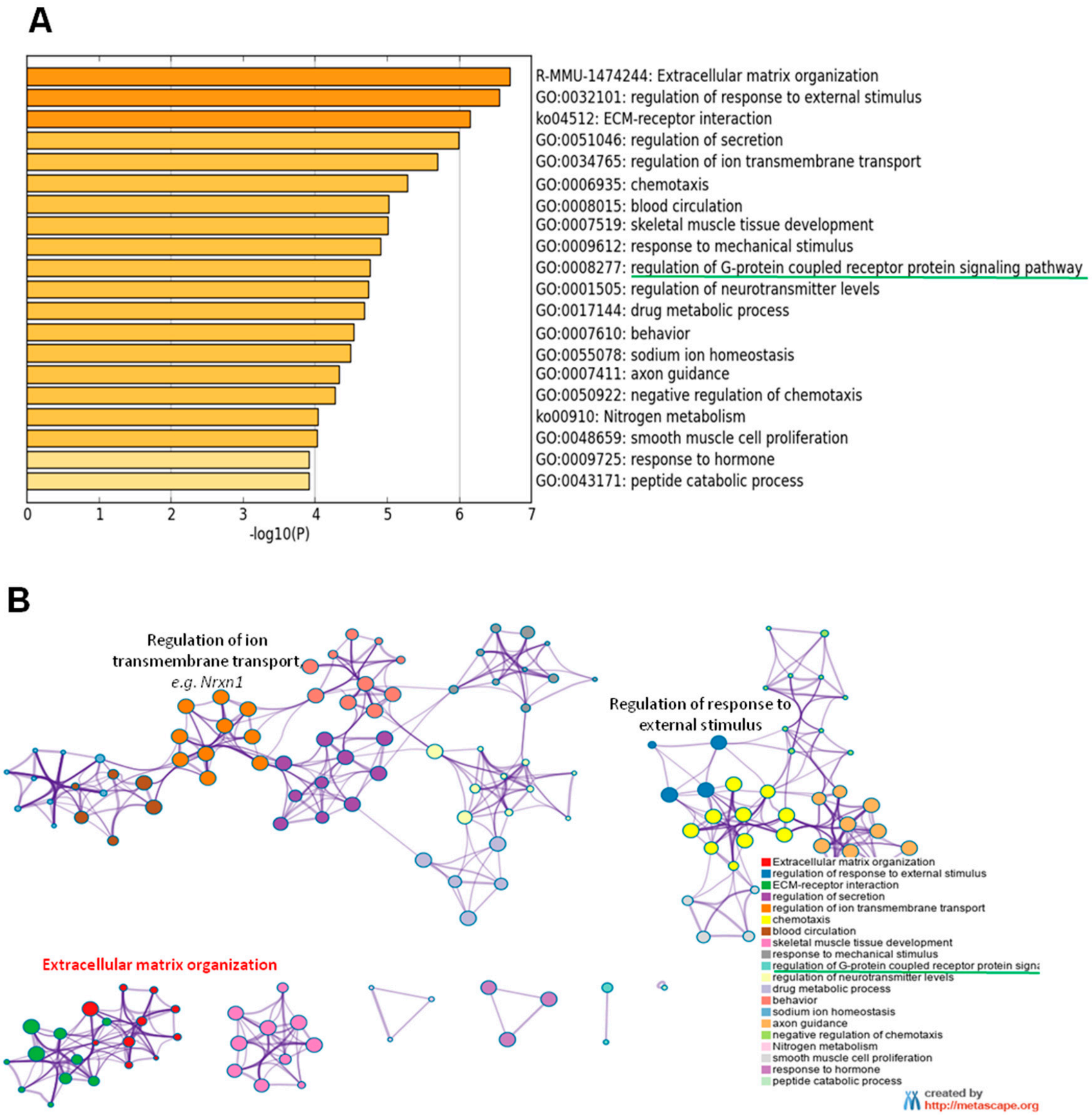

Figure S3. Overview of enriched clusters in GO terms obtained from RNA-seq analysis of down-regulated mRNA expression in Tspo mutated MA-10 cells. Statistically significant enriched terms (GO/KEGG terms and/or canonical pathways) were identified using Metascape (http://metascape.org). Accumulative hypergeometric p-values and enrichment factors were calculated and used for filtering. Remaining significant terms were hierarchically clustered into a tree based on Kappa statistical similarities among gene memberships. A Kappa score of 0.3 was applied as the threshold to cast the tree into term clusters. We selected a term with the lowest p-value as the representative term for each cluster and show the p-values of all clusters in the bar graphs. A. Cluster analysis for down-regulated genes under TSPO deficiency. B. Enriched pathways from the down-regulated mRNA expression in *Tspo* muted MA-10 cells. Gene-set clustering maps were generated using MetScape 3 for Cytoscape. The most significant shifting was the down-regulation of extracellular matrix organization (ECM) and ECM-receptor interaction. Other down-regulated genes, e.g. Nrxn1, are involved in ion transmembrane transport, G-protein coupled receptor signaling pathway, etc.

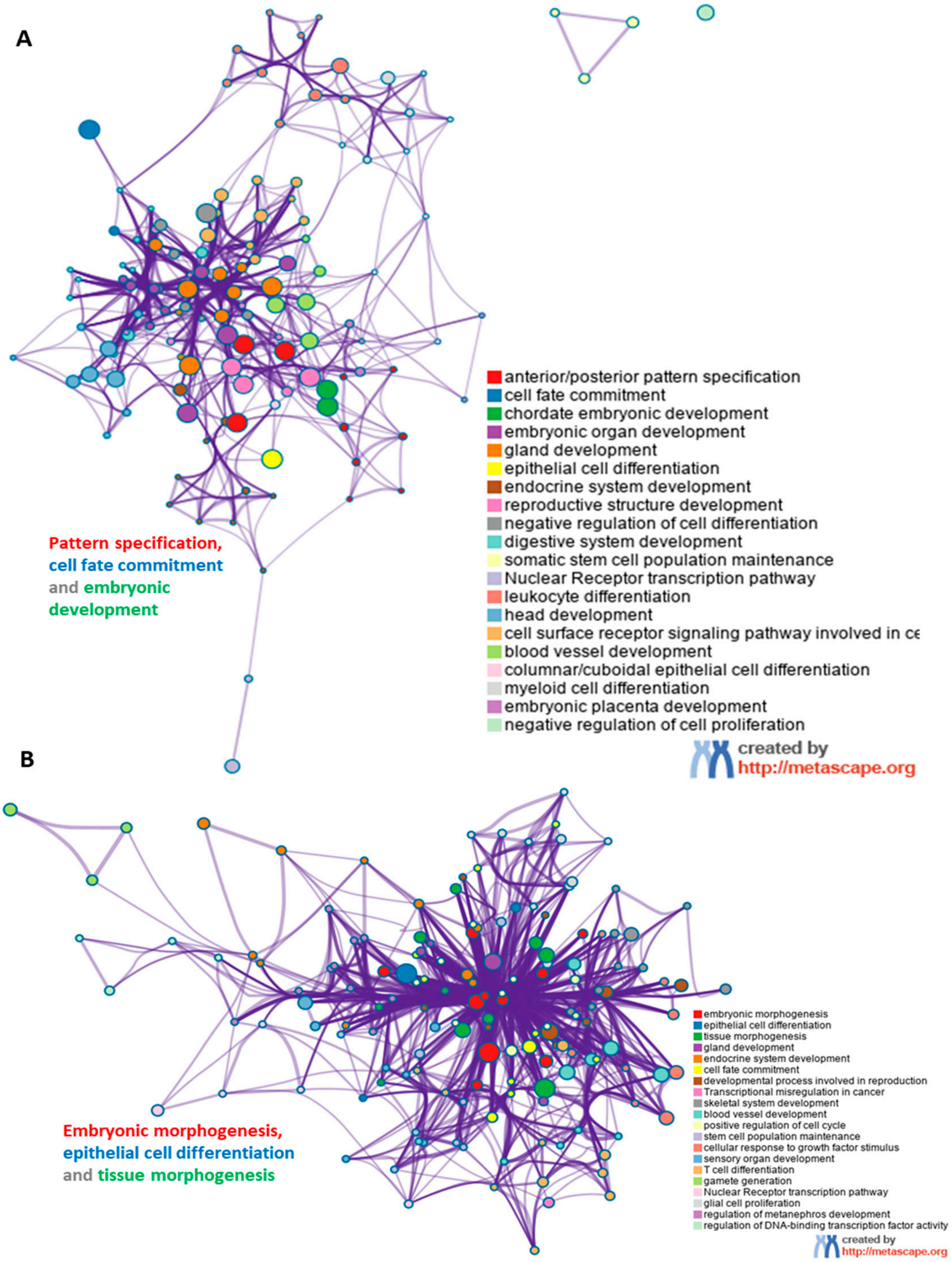

Figure S4. An UP (A) and DOWN (B) regulated gene network due to the loss of TSPO. Both network layouts were identified statistically and enriched in terms, including GO/KEGG terms, canonical pathways, hall mark gene sets, etc. More specifically, each term is represented by a circle node, where its size is proportional to the number of input genes fall into that term, and its color represent its cluster identity (i.e., nodes of the same color belong to the same cluster). Terms with a similarity score > 0.3 are linked by an edge (the thickness of the edge represents the similarity score). The network is visualized with Cytoscape (v3.1.2) with "force-directed" layout and with edge bundled for clarity. One term from each cluster is selected to have its term description shown as label.
