# Supplementary material for: Mitochondrial TSPO Deficiency Triggers Retrograde Signaling in MA-10 Mouse Tumor Leydig Cells
Source: Int J Mol Sci. 2020 Dec 29;22(1):252. doi: 10.3390/ijms22010252 (PMC7795497; doi:10.3390/ijms22010252)
Supplement: Supplementary file 1 [file ijms-22-00252-s001.zip › ijms-991545-supplementary-Table S1.pdf]

**Table S1.Up-regulated mRNA expression from *Tspo* mutant MA-10 cells.**

1110002E22Rik
1110012J17Rik
1200009I06Rik
1300017J02Rik
1600010F14Rik
1600029D21Rik
1700001L19Rik
1700019N12Rik
1700024P03Rik
1700026D11Rik
1700027D21Rik
1700028E10Rik
1700030F18Rik
1700034P13Rik
1700037F24Rik
1700054N08Rik
1700057G04Rik
1810024B03Rik
2010012P19Rik
2010015L04Rik
2210010C17Rik
2310005A03Rik
2310016G11Rik
2310042D19Rik
2400009B08Rik
2610028H24Rik
2610035D17Rik
2610203C22Rik
2610316D01Rik
2810405K02Rik
3110099E03Rik
4632428N05Rik
4731419I09Rik
4921517L17Rik
4921530D09Rik
4930405P13Rik
4930407I10Rik
4930415O20Rik
4930417G10Rik
4930428E23Rik
4930430D24Rik
4930432E11Rik
4930432M17Rik
4930448H16Rik
4930455H04Rik
4930469G21Rik

4930473A06Rik
4930479D17Rik
4930480G23Rik
4930502E18Rik
4930503E14Rik
4930505K14Rik
4930511F01Rik
4930511I11Rik
4930513O06Rik
4930558K02Rik
4930570N18Rik
4930583H14Rik
4930588K23Rik
4930595D18Rik
4930598A11Rik
4931413I07Rik
4932412H11Rik
4933400A11Rik
4933403G14Rik
4933405D12Rik
4933407P14Rik
4933413G19Rik
4933422H20Rik
4933426D04Rik
4933432B09Rik
4933433C11Rik
5330437I02Rik
5330439K02Rik
5430402O13Rik
5730419I09Rik
5830416P10Rik
6030469F06Rik
6030471H07Rik
6330403K07Rik
6530402F18Rik
8430403D17Rik
8430427H17Rik
8430429K09Rik
9030617O03Rik
9030624J02Rik
9130017N09Rik
9130204K15Rik
9230116N13Rik
9330154K18Rik
9330159F19Rik
9430065F17Rik
9530003J23Rik

9830144P21Rik
9930013L23Rik
A130010J15Rik
A230052G05Rik
A230083N12Rik
A330049M08Rik
A330050B17Rik
A3galt2
A430010J10Rik
A430033K04Rik
A430078G23Rik
A430093F15Rik
A430110N23Rik
A530040E14Rik
A530076I17Rik
A630014C17Rik
A730036I17Rik
A730085A09Rik
A830018L16Rik
A830036E02Rik
A930007I19Rik
A930029G22Rik
AA467197
AI182371
AI427809
AI428936
AI836003
Abat
Abca4
Abcb4
Abhd14b
Abi3bp
Acaa1b
Ace2
Acmsd
Acot12
Acot3
Acot5
Acp5
Acrbp
Acsm3
Acss3
Actc1
Actl6b
Actl9
Actn2
Actn3

Acvrl1
Adam15
Adam19
Adam33
Adap2
Adcy1
Adcy10
Adcy5
Adcy7
Adh4
Adh6
Adm2
Adora3
Adprhl1
Aebp2
Afap1l1
Afap1l2
Aff3
Agbl2
Agt
Agtpbp1
Aif1
Aifm2
Aim2
Aire
Ak5
Akap6
Akna
Aldh3a1
Alox15
Alpl
Amh
Amotl1
Ampd1
Ampd3
Amt
Amtn
Amz1
Angel1
Ank3
Ankmy1
Ankrd6
Ano7
Ano9
Anxa9
Aox4
Ap1m2

Apobec3
Apol9b
Aqp9
Arg2
Arhgap33
Arhgap4
Arhgap40
Arhgef10
Arhgef10l
Arl5c
Arpm1
Arrdc1
Arrdc5
Arsg
Art1
Art5
Arvcf
Asb16
Asb2
Asnsd1
Asprv1
Atoh8
Atp1a2
Atp2b4
Atp7a
Atp9a
Atxn7l1
Avil
B930007M17Rik
BC018473
BC024582
BC035044
BC039771
BC051019
BC052486
BC060267
BC061194
Bach2
Basp1
Batf2
Bbs1
Bbs12
Bcorl1
Best1
Bglap
Bicc1
Bmper

Bpifb1
Bpifb5
Bpifb9a
Brsk1
Btn2a2
Bzrap1
C030005K06Rik
C030019I05Rik
C130026I21Rik
C1qtnf3
C1qtnf5
C230014O12Rik
C330021F23Rik
C530008M17Rik
C530028O21Rik
Cabp4
Cacna1d
Cacna1e
Cacna1g
Cacna1i
Cadps2
Calcoco2
Calr4
Caly
Camk2a
Camsap3
Camta1
Capn11
Capn12
Capn5
Capn8
Capn9
Car5a
Car6
Car7
Card14
Card9
Casp1
Casp4
Casq1
Ccbl1
Ccbp2
Ccdc103
Ccdc125
Ccdc13
Ccdc146
Ccdc33

Ccdc48
Ccdc85a
Ccdc87
Ccdc88c
Ccdc92
Ccr7
Cct6b
Cd180
Cd209c
Cd209f
Cd209g
Cd247
Cd5
Cd74
Cd79a
Cd80
Cdc42ep3
Cdcp1
Cdh19
Cdh22
Cdk15
Ceacam10
Ceacam16
Ceacam19
Ceacam3
Cebpe
Celsr2
Cend1
Ces4a
Chmp4c
Chrnb2
Chrne
Chst12
Ciita
Cish
Ckmt1
Cldn10
Cldn2
Cldn27
Clip3
Clmn
Clps
Clta
Cmklr1
Cmtm2a
Cnih2
Cntfr

Cntnap5b
Col11a2
Col1a1
Col2a1
Col3a1
Col4a2
Col5a2
Col6a2
Col6a4
Cox6a2
Cox7b2
Cpsf4l
Cr2
Crb1
Crb2
Creb3l3
Crocc
Crxos1
Crygd
Cryge
Csrp1
Cst13
Cstl1
Ctsf
Cux2
Cuzd1
Cx3cl1
Cxx1c
Cyfip2
Cyp2t4
Cyp3a13
Cys1
D030055H07Rik
D130043K22Rik
D430040D24Rik
D630032N06Rik
D630042P16Rik
D6Mm5e
D730039F16Rik
D730045A05Rik
D930007P13Rik
Dab2ip
Dbn1
Dcdc2a
Dcp1b
Dcst1
Ddx25

Def6
Defb42
Dennd2d
Dennd4b
Dgat2
Dhrs9
Disp2
Dlx2
Dmrtc1a
Dmrtc2
Dnahc10
Dnaja4
Dnajb4
Dnase1l2
Dnase1l3
Dnhd1
Dnmt3l
Dock2
Dock4
Dok2
Dok3
Dpep1
Dpep2
Dpp4
Dppa2
Dpy19l1
Dsc1
Dsg1a
Dst
Dtx1
Dusp22
Dusp27
Dux
Dyrk4
Dzip1l
E030010N08Rik
E030025P04Rik
E130018N17Rik
E230008N13Rik
Eci3
Efna4
Efnb3
Efs
Egfl6
Ehf
Elf3
Elfn2

Elmod3
Emb
Emid1
Emid2
Emilin1
Eml5
Endou
Eng
Eno4
Entpd1
Entpd2
Epb4.9
Epha4
Ermap
Ern2
Erv3
Evx2
Exoc3l2
F13b
F730035M05Rik
F830208F22Rik
Fa2h
Faim2
Fam107b
Fam129c
Fam163b
Fam169b
Fam178b
Fam187a
Fam189b
Fam196b
Fam198a
Fam24a
Fam65c
Fam69c
Fam70b
Fam71d
Fam71f1
Fam71f2
Fam73a
Fam78a
Fam83a
Fam83c
Fam83f
Fam83h
Fap
Farp1

Fat3
Fbln5
Fbxl13
Fbxl2
Fbxo15
Fbxo24
Fcamr
Fcer1g
Fcgr1
Fcgr2b
Fermt1
Fgd3
Fgd5
Fgf21
Fgf9
Fgfr1
Fgfr3
Fgr
Fhl1
Fmnl1
Fmnl3
Fmr1nb
Fndc7
Folr1
Foxf1a
Frmd4a
Frmd7
Fry
Fscn1
Fstl1
Fut1
Fxyd7
G530011O06Rik
Gabrr1
Gal3st4
Garnl3
Gas1
Gata2
Gbgt1
Gcat
Gdf5
Gdpd3
Gfap
Gfi1b
Gfod2
Gfra2
Gfra4

Ggh
Ggt6
Gh
Ghr
Gimap6
Gipc2
Gja8
Gk5
Glis1
Glra4
Gm10109
Gm10134
Gm10253
Gm10258
Gm10354
Gm10373
Gm10433
Gm10474
Gm10517
Gm10575
Gm10606
Gm10660
Gm10715
Gm10716
Gm10717
Gm10718
Gm10719
Gm10722
Gm10731
Gm10800
Gm10801
Gm10804
Gm10982
Gm11019
Gm1110
Gm11167
Gm11292
Gm11415
Gm11437
Gm11525
Gm11532
Gm11616
Gm11635
Gm11683
Gm11716
Gm11730
Gm11884

Gm12052
Gm12056
Gm12150
Gm12153
Gm12199
Gm12228
Gm12280
Gm12405
Gm12473
Gm12511
Gm12798
Gm12976
Gm12992
Gm13032
Gm1305
Gm13054
Gm13078
Gm13083
Gm13090
Gm13101
Gm13203
Gm13227
Gm13235
Gm13242
Gm13334
Gm13373
Gm13387
Gm13404
Gm13562
Gm13660
Gm13752
Gm13778
Gm13782
Gm13783
Gm13807
Gm13830
Gm13842
Gm13869
Gm13872
Gm13874
Gm13943
Gm14002
Gm14019
Gm14025
Gm14047
Gm14102
Gm14280

Gm14302
Gm14319
Gm14320
Gm14391
Gm14486
Gm14597
Gm14641
Gm14703
Gm14820
Gm15070
Gm15135
Gm15348
Gm15398
Gm15418
Gm15419
Gm15518
Gm15523
Gm15525
Gm15541
Gm15548
Gm15582
Gm15612
Gm15614
Gm15640
Gm1568
Gm15729
Gm15759
Gm15775
Gm15788
Gm15856
Gm15894
Gm15958
Gm16022
Gm16028
Gm16029
Gm16031
Gm16130
Gm16150
Gm16183
Gm16191
Gm16214
Gm16240
Gm16267
Gm16306
Gm16320
Gm16321
Gm16333

Gm16343
Gm16506
Gm16532
Gm16565
Gm16576
Gm16628
Gm16630
Gm16743
Gm16831
Gm16869
Gm16956
Gm16994
Gm17019
Gm17033
Gm17041
Gm17067
Gm17106
Gm17135
Gm17189
Gm17359
Gm17370
Gm17445
Gm17509
Gm17535
Gm17589
Gm17623
Gm17673
Gm17718
Gm17728
Gm19980
Gm20489
Gm216
Gm2381
Gm3892
Gm3940
Gm4181
Gm4221
Gm4262
Gm4419
Gm4694
Gm4794
Gm4795
Gm4846
Gm4956
Gm4963
Gm5089
Gm514

Gm5415
Gm5475
Gm5624
Gm5699
Gm5897
Gm6133
Gm6288
Gm648
Gm6484
Gm6526
Gm6772
Gm684
Gm6871
Gm70
Gm711
Gm7489
Gm7714
Gm805
Gm8126
Gm8247
Gm8439
Gm9725
Gm9732
Gm9873
Gm9918
Gm9920
Gm9953
Gm9989
Gmpr
Gnal
Gnaz
Gngt2
Gp6
Gpa33
Gpc3
Gpnmb
Gpr133
Gpr150
Gpr157
Gpr158
Gpr160
Gpr17
Gpr20
Gpr56
Gpsm3
Greb1l
Grhl2

Grid2ip
Grin1
Grin2c
Grip2
Grrp1
Gucy2g
Gypa
H1foo
H2afy
H2afy2
Hapln3
Hapln4
Has2
Hck
Hcls1
Hcrt
Hdac7
Hdgfrp3
Heatr7b1
Hecw2
Hemt1
Hipk4
Hmcn2
Hnf4a
Hnrnpf
Hoxa3
Hoxa7
Hoxb3
Hoxb3os
Hrh3
Hrk
Hs3st1
Hsd11b1
Hsd17b3
Hsf5
Hspb2
Hspb8
Htr1f
Htra1
Htra3
Hunk
Hvcn1
Icam2
Ifi203
Ifi27l2b
Ifitm1
Ifitm7

Ifltd1
Igdcc3
Igf1
Igfbp4
Igfbp5
Igfn1
Igsf8
Ikzf3
Il12rb1
Il15
Il15ra
Il17f
Il17re
Il1a
Il1b
Il1rap
Il28a
Il2rg
Il31ra
Il34
Impg2
Inadl
Inhbb
Inhbc
Irak2
Irf6
Irf8
Irs2
Isl2
Islr2
Itga10
Itga11
Itga7
Itgad
Itgae
Itgax
Itgb1bp3
Itgb4
Itgb8
Iyd
Jag2
Kazn
Kbtbd12
Kcna1
Kcnab2
Kcnb1
Kcne2

Kcng4
Kcnh4
Kcnip2
Kcnj10
Kcnk15
Kcnk4
Kcnk7
Kcnn1
Kcnn2
Kcnn4
Kcnq4
Kcns3
Kcp
Kdm2b
Kdm5b
Kif9
Kiss1
Klb
Klc3
Klf1
Klf15
Klhdc8a
Klhl10
Klhl32
Klk1
Klk15
Klk1b1
Klk1b16
Klk1b21
Klk1b22
Klk1b24
Klk1b26
Klk1b27
Klk1b3
Klk1b4
Klk1b5
Klk1b8
Klrb1c
Krt23
Krt78
Krt82
L1cam
L3mbtl1
Lag3
Lair1
Lama2
Lamb3

Lasp1
Lbx2
Lcn2
Ldha
Ldhc
Lefty1
Lgals3bp
Lgals4
Lgals7
Lgals9
Lifr
Limk2
Lims2
Lipk
Lmo1
Lmod1
Lmtk3
Loxl4
Lpar5
Lpin1
Lrfn3
Lrguk
Lrrc48
Lrrc4b
Lrrc50
Lrrc61
Lrrc67
Lrrc7
Lrrc8a
Lrrc8d
Lsamp
Luzp4
Ly6g5c
Ly86
Lypd4
Lyrm7
Macrod2
Magea10
Mageb2
Mamdc2
Maml2
Manba
Mapt
Mast1
Mbd3l2
Mbp
Mcart6

Mcf2l
Mettl11b
Mettl21a
Mfap2
Mfap3l
Mfap5
Mfhas1
Mfi2
Mfrp
Mfsd2a
Mgll
Micalcl
Mid1ip1
Miox
Mlc1
Mmp28
Mmp9
Mogat2
Mospd4
Mprip
Mpv17l
Mras
Msgn1
Mst1
Mtl5
Mtus2
Muc15
Mybpc1
Myh11
Mylk
Myo15b
Myo18a
Myo1g
Myo1h
Myo5c
Myoz1
Mypn
Naalad2
Nacc2
Naip1
Nanos3
Nbeal2
Ncf1
Nctc1
Nedd4l
Neurl1a
Nfatc4

Nfe2
Nfe2l3
Nhedc1
Nhlh1
Ninj2
Nlgn3
Nlrp12
Nlrp4f
Nod1
Nova1
Nox1
Nphs1
Nppc
Npr2
Nr1i2
Nr1i3
Nr2f2
Nrap
Nrg4
Nrip2
Nrp2
Nrxn2
Nt5c3l
Ntn5
Ntng2
Nup210l
Nxnl1
Nyx
Oas3
Ocln
Odf3b
Odz4
Olfm1
Olfr1029
Olfr1030
Olfr18
Olfr287
Olfr39
Olfr750
Olfr872
Olfr873
Omp
Onecut3
Oprl1
Orai2
Orm1
Orm2

Osbpl10
Otoa
Otop1
Otop2
Otos
Otub2
Ovol1
P2rx1
P2rx6
P2rx7
P2ry6
Padi4
Pah
Pak6
Palld
Palmd
Pam
Panx2
Papss2
Paqr5
Pate2
Patz1
Pcdh10
Pcdhb1
Pcdhb2
Pcdhb3
Pcdhb5
Pcnxl2
Pcsk6
Pde11a
Pde2a
Pde6g
Pde6h
Pdgfa
Pdgfra
Pdgfrb
Pdzd3
Pdzd4
Pdzd7
Pecam1
Pemt
Pet2
Pex26
Pfn4
Pgls
Phf23
Phkg1

Phxr4
Phyhd1
Pi15
Pik3c2b
Pik3ip1
Pilrb1
Pilrb2
Pion
Pitpnm3
Pkd2l2
Pla1a
Pla2g10
Pla2g1b
Pla2g2e
Pla2g3
Pla2g4b
Plat
Plb1
Plcd4
Plch1
Plch2
Plekhb1
Plekhg1
Plk5
Plp1
Plxna4
Pmaip1
Pmel
Pmfbp1
Pnp2
Podxl2
Popdc2
Pparg
Ppef1
Ppef2
Ppfia4
Ppfibp2
Ppip5k1
Ppp1r13b
Ppp1r1b
Ppp2r2c
Ppp3ca
Pramef6
Prex1
Prkab2
Prkar1b
Prkcc

Prkcz
Prnd
Proca1
Prodh
Prr13
Prr15l
Prr19
Prrx1
Prss32
Prss35
Prss36
Prss57
Prx
Psd
Psd2
Psd4
Psg16
Psg29
Psma8
Pstpip1
Ptch2
Ptgds
Ptgfr
Ptgir
Pth1r
Ptprr
Ptpru
Pvrl4
Pydc4
Pzp
RP23
RP24
Rab20
Rab25
Rap1gap
Rasl11b
Rassf5
Rbms3
Rbpjl
Rdh12
Rdh16
Rdm1
Reln
Reps2
Rfx4
Rgag1
Rgs3

Rgs5
Rhbg
Rhox2a
Rhox2d
Rhox2h
Rin1
Ripply1
Rnase10
Rnasel
Rnf114
Rnf123
Rnf135
Rnf26
Rnf43
Robo2
Rph3al
Rps6ka1
Rsph10b2
Rsph6a
Ryr2
Ryr3
S100a16
S100a4
S100g
S1pr1
Samd4
Sash1
Satb1
Scarf1
Scrn1
Sebox
Sec14l2
Sec14l4
Sec14l5
Sema3g
Sema5b
Serpinb7
Serpinb8
Serpinf1
Serpinh1
Sfpi1
Sgca
Sh2d6
Sh3gl3
Sh3kbp1
Sh3pxd2a
Sh3rf2

Shisa4
Shisa7
Shroom4
Siglec1
Siglece
Sim1
Slc11a1
Slc11a2
Slc14a1
Slc14a2
Slc15a3
Slc16a12
Slc16a4
Slc17a7
Slc18a1
Slc1a2
Slc1a7
Slc23a3
Slc25a45
Slc26a3
Slc28a1
Slc2a6
Slc38a11
Slc39a11
Slc39a5
Slc43a2
Slc47a2
Slc4a10
Slc4a9
Slc6a4
Slc7a14
Slc7a7
Slc9a3
Slco2b1
Slfn2
Slurp1
Smad6
Smagp
Snap91
Snhg11
Soat2
Socs1
Sod3
Sorl1
Sort1
Sox13
Sox6

Sp1
Sp6
Spaca3
Spata21
Spata7
Spdyb
Speer4c
Speer4d
Speer7
Spg7
Spib
Spink8
Spinlw1
Spint2
Spnb3
Spnb5
Spock2
Spon1
Sprr2a1
Src
Srcin1
Srgap3
Sspo
St6galnac1
Stap2
Stat5a
Stbd1
Stc1
Stk32b
Stx1b
Stxbp2
Styxl1
Sulf1
Sulf2
Sult5a1
Sult6b1
Susd4
Sv2c
Sybu
Sycp3
Syn1
Syne1
Syt7
Sytl4
Tac2
Taf6
Tanc1

Tbc1d10c
Tbc1d21
Tbc1d30
Tbkbp1
Tbx10
Tbxas1
Tcam1
Tceanc
Tdo2
Tdrd9
Tead4
Tex13a
Tex14
Tfpi
Tg
Tgm3
Tgm4
Thrsp
Thsd7a
Thsd7b
Tjp2
Tktl1
Tlr7
Tm6sf2
Tmc3
Tmeff2
Tmem132b
Tmem139
Tmem140
Tmem156
Tmem176b
Tmem179
Tmem190
Tmem216
Tmem229b
Tmem29
Tmem37
Tmem38b
Tmem44
Tmem56
Tmem61
Tmem79
Tmem92
Tmod4
Tmprss6
Tnfrsf13c
Tnfrsf17

Tnfrsf25
Tnfrsf26
Tnk2
Tnni1
Tnnt1
Tnxb
Tomm20l
Tpd52
Tprg
Traf5
Trim21
Trim30a
Trim30d
Trim34a
Trim38
Trim40
Trim43b
Trim66
Trpm5
Trpv2
Tsix
Tspan10
Tspan18
Tspan32
Tspan9
Ttc16
Ttc21a
Ttll13
Tuba3b
Tuba8
Tulp1
Tulp2
Txlnb
Ube2l3
Ube2l6
Ucp3
Uggt2
Ugt1a6a
Ugt1a6b
Ugt1a7c
Ugt2a3
Unc119
Unc13d
Unc45b
Unc80
Ush1g
Usp18

Usp46
Usp54
Vdr
Vezt
Vgll1
Vmn1r4
Vmn2r44
Vmo1
Vpreb3
Vwa3a
Wfdc3
Wnk2
Wnt5b
Wnt7b
Wnt8b
Wnt9a
Xaf1
Xcr1
Xirp1
Xist
Xkr5
Xkrx
Xlr4a
Xpnpep2
Xrra1
Ybx2
Yipf1
Ysk4
Zbtb32
Zdhhc14
Zdhhc19
Zfp114
Zfp133
Zfp365
Zfp366
Zfp423
Zfp454
Zfp462
Zfp518b
Zfp534
Zfp558
Zfp580
Zfp600
Zfp658
Zfp69
Zfp786
Zfp951

Zfyve28
Zic3
Zmynd10
Zscan4c
Zswim5
Zyg11a
