# Supplementary material for: Mitochondrial TSPO Deficiency Triggers Retrograde Signaling in MA-10 Mouse Tumor Leydig Cells
Source: Int J Mol Sci. 2020 Dec 29;22(1):252. doi: 10.3390/ijms22010252 (PMC7795497; doi:10.3390/ijms22010252)
Supplement: Supplementary file 1 [file ijms-22-00252-s001.zip › ijms-991545-supplementary-Table S2.pdf]

Table S2. Down-regulated mRNA expression from *Tspo* mutant MA-10 cells.

1110035M17Rik
1500009L16Rik
1700003M07Rik
1700008I05Rik
1700011E24Rik
1700011I03Rik
1700016P03Rik
1700026L06Rik
1700049E15Rik
1700061J23Rik
1700112E06Rik
1700113H08Rik
2010305A19Rik
2200002K05Rik
2610034M16Rik
2610109H07Rik
2610206C17Rik
2810403D21Rik
3200002M19Rik
3632451O06Rik
4732465J04Rik
4833427F10Rik
4921504A21Rik
4921528I01Rik
4930426D05Rik
4930480K15Rik
4930511M11Rik
4933430H15Rik
5031410I06Rik
5033411D12Rik
5730420D15Rik
6330439K17Rik
6820408C15Rik
9030025P20Rik
9130230L23Rik
A230005M16Rik
A230059L01Rik
A330040F15Rik
A530098C11Rik
AU019990
Aarsd1
Abcb9
Abcc2
Abcc9
Abcg5
Acat3

Acer2
Acsbg2
Acss1
Actr3b
Adarb1
Adcyap1
Aff2
Agap2
Agbl3
Agbl5
Agxt2
Ahcyl2
Ak8
Aldh3b2
Aldh8a1
Aldob
Alg13
Alpk3
Amdhd1
Amot
Ankrd2
Ankrd35
Ano1
Ano3
Anpep
Apcdd1
Apobec1
Apoc3
Apol8
Arhgap22
Arhgap28
Arhgap44
Arhgap9
Arhgef40
Arl11
Art3
Artn
Asgr1
Atat1
Atg13
Atp1a3
Atp8b5
Avpr1a
B230314M03Rik
BC016201
BC021767
BC023105

BC042782
BC048594
BC055111
Baalc
Bai2
Bche
Bean1
Bmp2
Bmpr1b
Brdt
Brsk2
Btbd3
Btbd9
Btg2
C1ql4
C3
C3ar1
C430014B12Rik
C4a
C4b
C630004H02Rik
Cacna1h
Cacna2d3
Cacnb2
Capn3
Car1
Car15
Car5b
Car8
Caskin1
Catsperg1
Catsperg2
Cblc
Cbr2
Cbs
Ccdc148
Ccdc154
Ccdc162
Ccdc37
Ccdc38
Ccdc64b
Ccdc88b
Cd109
Cd22
Cd244
Cd34
Cd7

Cda
Cdh13
Cdh16
Cdh17
Cdh9
Cdk14
Cdkl2
Cdkl3
Cdon
Ceacam2
Cela3b
Celf3
Celf6
Cerkl
Chchd6
Chdh
Chrd
Chrm5
Chrna4
Cilp
Cited4
Clca1
Clca3
Clca5
Clec11a
Clec16a
Clu
Cndp1
Cngb1
Cnksr1
Cntnap2
Col11a1
Col14a1
Col16a1
Col19a1
Col22a1
Col4a5
Col4a6
Col6a6
Col7a1
Cp
Cpa6
Cpe
Cps1
Cpt1c
Creg1
Crem

Crhr2
Crim1
Crisp1
Crisp3
Crispld2
Crx
Csf1r
Csf2ra
Csgalnact1
Csmd1
Csmd3
Csn1s1
Csnk1e
Cyp11b1
Cyp19a1
Cyp1a1
Cyp2c55
Cyp2d11
Cyp2f2
D030054H15Rik
D430001F17Rik
D7Ertd443e
Dchs1
Dcx
Ddah2
Defb19
Defb36
Dennd3
Des
Dgkk
Dlg4
Dlgap1
Dlgap4
Dmd
Dnahc2
Dnahc3
Dnahc5
Dnahc6
Dnahc9
Dnm1
Dpcr1
Dpp6
Dusp4
Dydc2
Dynlt1e
E030011O05Rik
E130114P18Rik

E330009J07Rik
Eaf2
Eda2r
Edil3
Edn1
Efcab4a
Efcab5
Efhb
Efhc1
Efr3b
Egr1
Egr2
Egr3
Elf5
Elk3
Enam
Enkur
Eno2
Entpd3
Etv4
Eya1
F5
Fabp2
Fah
Fam115c
Fam125b
Fam135b
Fam154b
Fam166b
Fam3c
Fam46a
Fam55d
Fam5c
Fank1
Fat2
Fblim1
Fbp1
Fbxl17
Fbxo2
Fcho1
Fez1
Fezf2
Fgf7
Flot1
Fmn2
Fosl1
Frmd4b

Frmpd1
Fsip1
Fstl5
Gabrb2
Gabrg1
Gck
Gcom1
Gdf7
Gem
Ggt5
Ggt7
Gif
Gipr
Gli1
Gm10220
Gm10308
Gm10471
Gm10497
Gm11127
Gm11210
Gm1123
Gm1141
Gm11754
Gm12530
Gm12860
Gm12861
Gm12865
Gm12949
Gm12963
Gm13052
Gm13070
Gm13073
Gm13096
Gm13147
Gm13154
Gm13165
Gm13225
Gm13388
Gm13522
Gm13713
Gm13762
Gm13857
Gm14074
Gm14257
Gm14341
Gm14526
Gm14978

Gm15396
Gm15512
Gm15639
Gm15883
Gm16043
Gm16120
Gm16140
Gm16193
Gm16212
Gm16271
Gm16548
Gm16587
Gm16702
Gm1673
Gm16799
Gm16853
Gm16962
Gm17040
Gm17120
Gm17507
Gm1979
Gm20449
Gm20481
Gm20482
Gm2155
Gm239
Gm3470
Gm4461
Gm4877
Gm4889
Gm4981
Gm5432
Gm5819
Gm5830
Gm5862
Gm597
Gm6034
Gm6657
Gm6996
Gm7168
Gm7347
Gm7361
Gm773
Gm8258
Gm830
Gm88
Gm973

Gm98
Gm9930
Gnb4
Gnrhr
Got1l1
Gpr113
Gpr115
Gpr137c
Gpr153
Gpr182
Gpr35
Gpr55
Grin2d
Grinl1a
Grk4
Gstm3
Gstm6
Gsx2
Hcrtr1
Hephl1
Hfm1
Hgfac
Hivep3
Hmgn3
Hmox1
Hnf1b
Hoxc4
Hs3st6
Hsd3b1
Hsf2bp
Hspa1a
Htr6
Hyal1
Ier3
Iigp1
Ikzf4
Il17rb
Il17rd
Il28ra
Il4ra
Inpp5d
Iqck
Irak4
Isg20
Itga2
Itga4
Itga8

Jph2
Jsrp1
Kalrn
Kcng3
Kcnu1
Kctd19
Khdc1a
Klf4
Klhl14
Klhl33
Klrc1
Krt81
Krt86
L3mbtl4
Lama3
Lama5
Lamc3
Lbp
Ldb3
Lepr
Lhcgr
Lhfpl1
Lhfpl5
Lingo2
Lmo2
Lrmp
Lrp1
Lrp8
Lrpprc
Lrrc26
Lrrc55
Lrrc6
Ly6i
Ly9
Lypd1
Lypd6
Maff
Magi2
Mapk15
Mapk8ip1
Masp1
Mat1a
Mb
Megf10
Met
Mfsd6
Micall2

Mmp12
Mmp13
Mmp19
Mmp27
Mmp8
Mpp2
Mpp3
Mrgprf
Msln
Msrb3
Mta3
Mtap7d2
Mthfd1l
Mtmr1
Mtmr11
Muc1
Myh7b
Myo18b
Myo7a
Naip6
Nalcn
Nccrp1
Ncf2
Ncrna00086
Neb
Nedd9
Nek10
Neurl3
Nhs
Nkain2
Nlrc3
Nme5
Nos1
Notch3
Notch4
Npm2
Nppb
Nr4a3
Nrd1
Nrtn
Nrxn1
Nsd1
Ntf5
Nudt16
Oasl2
Ociad2
Ogdhl

Olfr1000
Olfr1006
Olfr1012
Olfr1013
Olfr1014
Olfr1213
Olfr1246
Olfr154
Olfr370
Olfr419
Olfr456
Olfr457
Olfr853
Olfr933
Opn4
Otop3
P2rx2
P2rx5
P4htm
Padi2
Paqr6
Paqr8
Park2
Parp14
Pbx1
Pde4c
Pdlim5
Pdxk
Pebp4
Pigk
Pik3ap1
Pitx3
Piwil4
Pla2g5
Plac8
Plau
Plcxd1
Plcxd3
Pnma5
Poc1b
Podnl1
Ppard
Ppil6
Ppp1r3g
Pqlc1
Prickle1
Prl

Prl2c3
Prl7a2
Prl7b1
Prl7d1
Prlr
Procr
Prr9
Prrg4
Prrt1
Prrt4
Prss22
Prss23
Prss42
Ptger4
Ptplad2
Ptpn5
Ptpra
Ptx4
Rab15
Rab40b
Rac2
Ramp1
Rapgef3
Rarres2
Rasa3
Rasgrf2
Rasgrp4
Rassf4
Raver2
Rbm20
Rbm44
Relb
Rgs10
Rgs13
Rgs2
Rgs9
Rhov
Rims1
Rlbp1
Rltpr
Rnf157
Rnf180
Rnls
Rnpep
Robo1
Robo3
Rpgrip1

Rpl31
Rpl3l
Rps6ka2
Rps6ka5
Rsph9
Rtkn2
Runx2
Ryr1
Samd5
Scamp1
Scin
Scn4a
Sdc3
Sdcbp
Selp
Sema3a
Sema3d
Sema3f
Sema5a
Sema6b
Serpinb6c
Serpinb6d
Serpinb9c
Setd7
Sez6
Sftpc
Sh2d3c
Sh2d4b
Sh3bgr
Shc4
Siglecg
Sim2
Skint3
Slc12a2
Slc22a18
Slc23a1
Slc26a5
Slc2a9
Slc30a1
Slc35f4
Slc39a2
Slc4a4
Slc4a5
Slc5a8
Slc6a9
Slfnl1
Snai1

Sntb1
Snx24
Socs2
Spata13
Spata18
Specc1
Speer4a
Speer4b
Speg
Spic
Spna1
Spp1
Spred3
St14
St18
Stab2
Steap1
Steap3
Stim2
Stk36
Stk40
Styk1
Susd1
Susd2
Sv2a
Syngr3
Sytl1
Sytl3
Tagln
Tas2r135
Tbc1d14
Tbc1d16
Tceal8
Tcp10b
Tcte2
Tead1
Tec
Tecrl
Tekt4
Tesc
Tex12
Tex15
Tex19.1
Tex19.2
Tgm5
Tgm6
Tgs1

Thbs1
Thbs2
Thsd4
Tiam2
Tle2
Tll2
Tlr4
Tmcc2
Tmem119
Tmem164
Tmem173
Tmem178
Tmem181a
Tmem184a
Tmem22
Tmem232
Tmem40
Tmem8b
Tmprss11bnl
Tmprss7
Tmsb15b2
Tmsb15l
Tnfaip6
Tnnt3
Trcg1
Trdn
Trfr2
Trim63
Trim69
Trim72
Triobp
Tro
Trpa1
Tspan17
Ttc39c
Ttll9
Tubb2b
Tulp4
Uaca
Uba7
Ugdh
Ugt2b36
Ugt2b5
Unc13b
Vgll3
Vill
Vit

Vmn1r66
Vmn2r13
Vmn2r31
Vmn2r33
Vmn2r79
Vmn2r97
Vsnl1
Vwa2
Vwa5b2
Wbscr27
Wdr52
Wisp2
Wiz
Xkr4
Xkr9
Xlr3a
Xrcc4
Zan
Zbtb38
Zdbf2
Zdhhc9
Zfp277
Zfp300
Zfp37
Zfp516
Zfr2
