# Supplementary material for: Mitochondrial TSPO Deficiency Triggers Retrograde Signaling in MA-10 Mouse Tumor Leydig Cells
Source: Int J Mol Sci. 2020 Dec 29;22(1):252. doi: 10.3390/ijms22010252 (PMC7795497; doi:10.3390/ijms22010252)
Supplement: Supplementary file 1 [file ijms-22-00252-s001.zip › ijms-991545-supplementary-Table-S3.pdf]

Table S3. Transcription factors (TFs) affected by the loss of TSPO.

Up-regulated	Down-regulated
Aire	Arid5b
Arnt	Arntl
Ascl2	Barx2
Bach2	Batf
Barx1	Clock
Batf3	Crem
Cdx1	Crx
Cdx2	**Egr1**
Cebpe	**Egr3**
Cux2	Etv2
Dlx3	Etv4
Egr4	Etv5
Ehf	Fli1
Elf2	**Fosb**
Elf3	**Fosl1**
Elf5	Foxa3
Esrrg	Gata5
Ets2	Gli1
Foxa2	Hes1
Foxp2	Hnf1b
Foxp3	Hoxa13
Gata1	Irf7
Gfi1b	Klf4
Gli2	Maf
Gli3	Mafb
Glis3	Nfya
Grhl2	Nkx2
Hesx1	Nobox
Hey2	Nr1d2
Hmga2	Nr1h4
Hnf4a	Nr4a3
Hoxa5	Olig2
Hoxa7	Onecut2
Hoxb6	Ovol2
Hoxd10	Pax2
Hoxd13	Pbx1
Insm1	Plag1
**Irf8**	Pou6f1
Klf1	Prdm9
**Klf15**	Prrx2
Klf5	Rarb
Lef1	Rfx2
Mecom	Runx1
Mef2a	Runx2

Meis1	Sox4
Msgn1	Taf1
Msx3	Tal1
Myb	Tbp
Myog	Tgif1
Nanog	
Neurog2	
Nfatc4	
Nfe2	
**Nfkb1**	
Nkx3	
Nkx6	
Nr1d1	
Nr1i2	
Nr2f1	
Nr2f2	
Nr3c2	
Nr5a2	
Otx1	
Pou1f1	
Pou2f1	
Prdm14	
Prdm16	
Prop1	
Prrx1	
Relb	
Rfx3	
Runx3	
Rxrg	
Snai2	
Sox10	
Sox17	
Sox3	
Sox5	
Sox9	
Sp4	
Sp5	
Sp7	
Stat4	
Tbx21	
Tcf7	
Tcf7l1	
Tlx1	
Wt1	
Zfp57	
Zic3	

*the same family of TFs has been validated using real-time PCR or highlighted in the Fig S6 (Red, up-regulated; green, down-regulated)
